# A potential role for RNA aminoacylation prior to its role in peptide synthesis

**DOI:** 10.1073/pnas.2410206121

**Published:** 2024-08-23

**Authors:** Aleksandar Radakovic, Anna Lewicka, Marco Todisco, Harry R. M. Aitken, Zoe Weiss, Shannon Kim, Abdullah Bannan, Joseph A. Piccirilli, Jack W. Szostak

**Affiliations:** ^a^HHMI, Department of Chemistry, The University of Chicago, Chicago, IL 60637; ^b^Department of Genetics, Harvard Medical School, Boston, MA 02115; ^c^Department of Biochemistry and Molecular Biology, The University of Chicago, Chicago, IL 60637; ^d^HHMI, Department of Molecular Biology and Center for Computational and Integrative Biology, Massachusetts General Hospital, Boston, MA 02114; ^e^Department of Chemistry and Chemical Biology, Harvard University, Cambridge, MA 02138; ^f^Department of Molecular and Cellular Biology, Harvard University, Cambridge, MA 02138

**Keywords:** aminoacylation, T-loop, ribozymes, RNA World

## Abstract

Coded ribosomal peptide synthesis could not have evolved unless its sequence and amino acid–specific aminoacylated tRNA substrates already existed. We therefore wondered whether aminoacylated RNAs might have served some primordial function prior to their role in protein synthesis. Here, we show that specific RNA sequences can be nonenzymatically aminoacylated and ligated to produce amino acid–bridged stem-loop RNAs. We used deep sequencing to identify RNAs that undergo highly efficient glycine aminoacylation followed by loop-closing ligation. The crystal structure of one such glycine-bridged RNA hairpin reveals a compact internally stabilized structure with the same eponymous T-loop architecture that is found in many noncoding RNAs, including the modern tRNA. We demonstrate that the T-loop-assisted amino acid bridging of RNA oligonucleotides enables the rapid template-free assembly of a chimeric version of an aminoacyl-RNA synthetase ribozyme. We suggest that the primordial assembly of amino acid–bridged chimeric ribozymes provides a direct and facile route for the covalent incorporation of amino acids into RNA. A greater functionality of covalently incorporated amino acids could contribute to enhanced ribozyme catalysis, providing a driving force for the evolution of sequence and amino acid–specific aminoacyl-RNA synthetase ribozymes in the RNA World. The synthesis of specifically aminoacylated RNAs, an unlikely prospect for nonenzymatic reactions but a likely one for ribozymes, could have set the stage for the subsequent evolution of coded protein synthesis.

## Significance

Prebiotically plausible nonenzymatic aminoacylation of RNA has been an elusive but key requirement for studying the origin of translation. Here, we show that even inefficient, chemical RNA aminoacylation can be harnessed to assemble chimeric amino acid–bridged RNA loops in high yield. The RNA loop architecture that is assembled most efficiently has the characteristics of a T-loop, a common structural element found in tRNA, rRNA, and many other noncoding RNAs. The T-loop-mediated assembly of chimeric RNA can lead to rapid assembly of a chimeric aminoacyl-RNA synthetase ribozyme without requiring a complementary template. Our findings connect nonenzymatic aminoacylation to a common RNA structural element and show that aminoacylation can facilitate the assembly of RNA-based catalysts.

In all extant life, amino acids are covalently attached to tRNAs by aminoacyl-tRNA synthetase enzymes to generate the substrates for ribosomal translation. Because RNA- and amino acid-specific aminoacylation is necessary for coded protein synthesis, aminoacyl-RNA synthetase ribozymes likely predated the ribosome (1–3). Many RNA aminoacylating ribozymes have been isolated by laboratory evolution, supporting this possibility (4–7). However, their evolution in the RNA World presupposes a selective advantage for the synthesis of aminoacylated RNAs. We have previously demonstrated that the enhanced reactivity of aminoacylated RNAs can accelerate template-directed ligation and facilitate the assembly of active ribozymes composed of chimeric oligomers of RNA and amino acids (8, 9). However, nonenzymatic RNA aminoacylation is inefficient (4, 10–13). In the absence of a significant background level of RNA aminoacylation, it is difficult to imagine a selection pressure that could drive the evolution of ribozymes that would enhance RNA aminoacylation. Amino acids are thought to have been widely available on the early Earth (14–16), and multiple prebiotic amino acid activation pathways have been explored (11, 17–21) but due to the poor nucleophilicity of the 2′,3′-diol of RNA (22), activated amino acids tend to either hydrolyze or polymerize into random peptides before RNA aminoacylation can occur (11, 12, 17, 18, 23). The inefficient synthesis of aminoacylated RNA is further compounded by the rapid hydrolysis of aminoacyl esters (24–27), resulting in low steady-state levels of aminoacylated RNA even with repeated amino acid activation.

Induced proximity is an effective strategy for enabling aminoacylation to compete with hydrolysis and peptide formation. For example, in a nicked duplex, aminoacylated RNA can be made in 15 % yield via aminoacyl transfer from a 5′-phosphocarboxy anhydride to the 2′,3′-diol (28). Nicked hairpin loop architectures can also facilitate aminoacyl transfer chemistry, with a yield that is dictated by the sequence of the RNA loop (29). However, this route to aminoacyl-RNA synthesis relies on preformed 5′-phosphocarboxy anhydrides, which are even more labile than aminoacyl esters (12, 18). Beginning instead with preformed 5′-phosphoramidates, where the N-terminus of an amino acid is linked to a 5′-phosphate, the 2′,3′-diol can efficiently capture the amino acid carboxylate under activating conditions to form chimeric, amino acid-bridged hairpin loops. Aminoacyl-RNA can then be retrieved after acid hydrolysis of the phosphoramidate linkage (30), but how early life could exploit such a multistep synthesis of aminoacylated RNA is not clear.

**Fig. 1.**
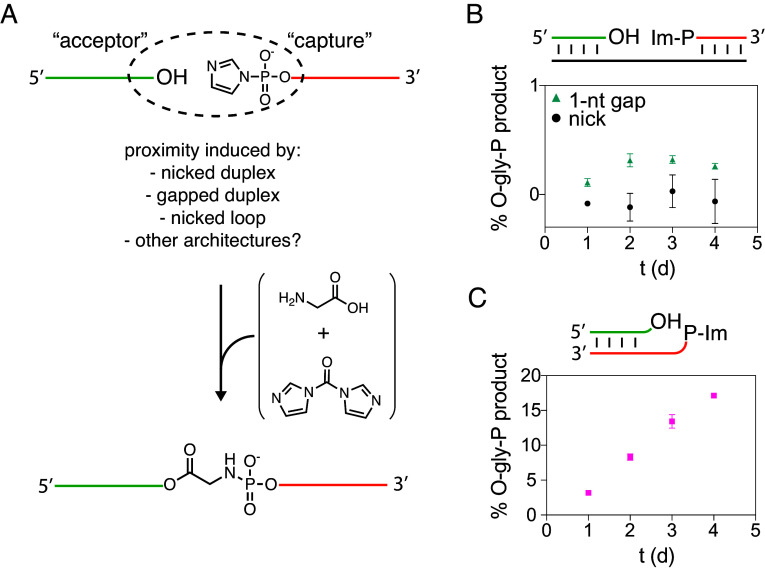
Aminoacylation capture strategy. (*A*) An acceptor RNA and a capture RNA that is activated as a 5′-phosphorimidazolide (Im-P) react with a CDI-activated glycine to form an amino acid bridged (O-gly-P in this case) product. (*B*) Low yield of aminoacylation capture in a nicked duplex (black circles) or a duplex with a one-nucleotide gap (teal triangles). (*C*) Aminoacylation capture by a nicked stem-loop construct. All reactions were performed in technical triplicates in 100 mM HEPES pH 8, 5 mM MgCl_2_, 10 µM oligonucleotides, 50 mM premixed CDI + gly added in 12.5 mM increments every 24 h at 0 °C. See *SI Appendix*, Table S3 for the exact sequences used.

Here, we demonstrate that certain RNA sequences can be nonenzymatically aminoacylated and then captured by the formation of amino acid–bridged RNA stem-loops in near-quantitative yield. This process of activated amino acid capture occurs via a two-step reaction pathway in which an activated amino acid carboxylate reacts with the 3′-terminus of an acceptor oligonucleotide, and the amine reacts with the 5′-phosphorimidazolide of a downstream capture strand (Fig. 1*A*). The second step leverages the spatial proximity afforded by the RNA secondary structure to accelerate the ligation. We used deep sequencing to identify 3′-overhang sequences that capture activated glycine efficiently. We solved the structure of one such glycine-bridged stem-loop by X-ray crystallography and found that the structural features necessary for loop-closing ligation closely match those found in T-loops, structures that are conserved in noncoding RNAs, such as tRNA and rRNA. We then demonstrated that the T-loop capture of activated glycine can be used to assemble a functional, chimeric aminoacyl-RNA synthetase ribozyme in high yield and without the need for a template. Our work outlines a sequence of plausible reactions that leverages inefficient nonenzymatic RNA aminoacylation to rapidly produce an RNA-based catalyst that facilitates efficient RNA aminoacylation. Ribozymes facilitating aminoacylation would be crucial for assembling a variety of other chimeric ribozymes that might require specific bridging amino acids to be positioned within specific sequence contexts. We suggest that the selective advantage resulting from the ability to assemble chimeric ribozymes with precisely positioned internal amino acids, an ability that is difficult to ascribe to nonenzymatic aminoacylation processes, could have favored the evolution of sequence and amino acid–specific aminoacyl-RNA synthetase ribozymes, thus setting the stage for the evolution of coded peptide synthesis.

## Results

### Proximity-Based Aminoacyl-RNA Capture by Nicked Loops.

We have focused on glycyl-imidazolide as a prebiotically plausible activated amino acid. Glycyl-imidazolide is generated by the reaction of glycyl-*N*-carboxyanhydride (gly-NCA) with imidazole, and gly-NCA can in turn be generated from glycine in the presence of carbonyl sulfide (17). For convenience, we used *N, N*′-carbonyldiimidazole (CDI) to prepare glycyl-imidazolide from glycine (*SI Appendix*, Scheme S1) (31). Our preliminary experiments suggested very low yields of 2′(3′)-aminoacylated RNA using CDI-activated glycine (*SI Appendix*, Figs. S1 and S2), consistent with earlier literature that detected little or no product of the reaction of activated amino acids and RNA (13, 32). Because the aminoacyl ester is greatly stabilized in amino acid-bridged RNA synthesized from preaminoacylated RNA (8, 27), we tested whether in situ glycylation could be captured by a nicked duplex with an activated 5′-phosphate. To our surprise, we could not detect any glycine-bridged product using either nicked or gapped duplexes (Fig. 1*B* and *SI Appendix*, Fig. S3). Serendipitously, while using the Flexizyme ribozyme to aminoacylate the 3′-terminus of a 5′-(2-methylimidazole)-activated oligonucleotide, we found that the nicked loop architecture allows for both efficient aminoacylation and amino acid-bridging ligation (*SI Appendix*, Fig. S4). Incubating a 5′-activated nicked loop based on the P2 stem-loop found in the dFx Flexizyme (6) with CDI-activated glycine, we were gratified to observe ligated glycine-bridged product in 17% initial and 30% optimized yield (Fig. 1*C* and *SI Appendix*, Fig. S5). To establish the prebiotic relevance of our chemistry, we presynthesized gly-NCA and demonstrated that glycyl-imidazolide is the activated species captured by the loop (*SI Appendix*, Scheme S1 and Fig. S6). Failure to form the ligated product after periodate oxidation of the RNA, rapid hydrolysis of the loop-closed product upon alkaline treatment, and high-resolution mass spectrometry were all consistent with a single glycyl ester linkage bridging the diol of the acceptor and the 5′-phosphate of the capture strand (*SI Appendix*, Fig. S7).

The stability to hydrolysis of the glycine-bridged loop at pH 8 far exceeded the stability of the same bridge in a duplex context, or a comparable bridge in a single-stranded construct (8) (*SI Appendix*, Table S1 and Fig. S8). This result suggested that the structure of the loop might serve to capture and thereby accumulate an otherwise labile aminoacylated RNA in a stable amino acid–bridged product. In addition to glycine, this particular nicked loop architecture captured activated Phe, Ala, and Leu to varying degrees and exhibited moderate stereoselectivity for the L enantiomer, hinting at a potential stereochemical selectivity mechanism based on the RNA architecture (*SI Appendix*, Fig. S9).

### Deep Sequencing Screen for Efficient Capture Sequences.

**Fig. 2.**
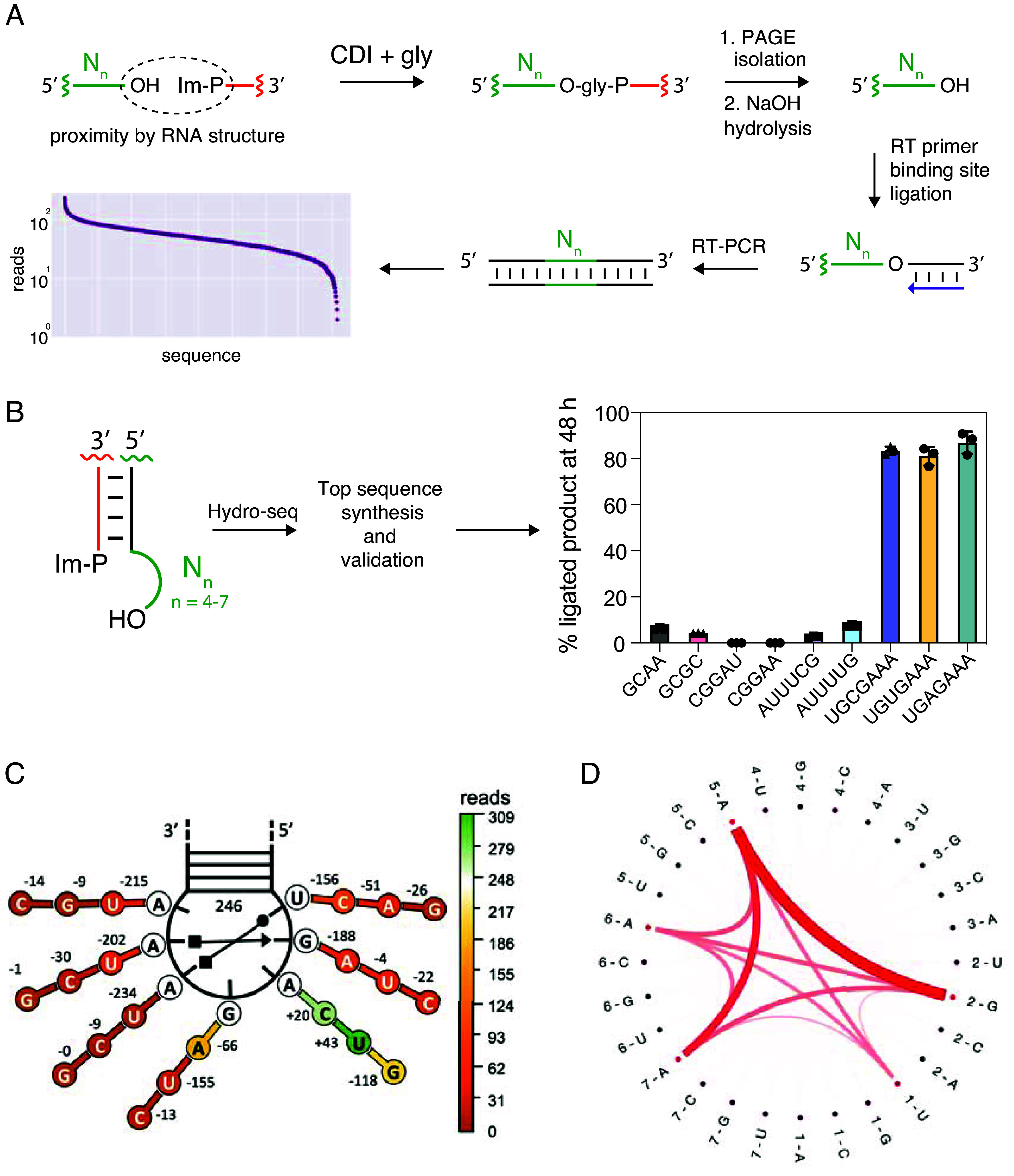
Sequencing assay for the identification of efficient capture sequences. (*A*) Schematic of the sequencing assay. A library of acceptor RNA strands consisting of a constant region followed by 4 to 7 randomized nucleotides was incubated with a complementary constant sequence capture strand bearing an imidazole-activated 5′-phosphate and then chemically aminoacylated with CDI and glycine. Base-pairing of the donor and acceptor strands generates a five base-pair stem with a 4 to 7 nucleotide 3′-overhang. Loop-closed products were purified by PAGE and the amino acid bridge selectively hydrolyzed to liberate the acceptor strand. Quantitative untemplated, enzymatic ligation of the RNA 3′-end to an oligonucleotide that acts as a primer binding site for reverse transcriptase allowed for cDNA synthesis, PCR amplification, and deep sequencing. (*B*) The capture reaction using the two or three sequences with the greatest number of reads from each stem-loop construct (4 to 7-nt overhangs on a double-stranded stem). The reaction was performed in technical triplicates in 100 mM imidazole pH 8, 5 mM MgCl_2_, 1 µM oligonucleotides, and 50 mM CDI + gly at 0 °C. (*C*) Substitution analysis based on the sequencing data. The change in the number of reads is shown for each nucleotide mutation of the 5′-UGAGAAA-3′ overhang independent of the other mutations. (*D*) Median interaction plot as computed from Shapley values in the top cluster. Thickness and color density represent the strength of interaction between pairwise features.

To identify the RNA 3′-overhang sequences that most efficiently capture activated amino acids, we designed a screen based on high-throughput sequencing (Fig. 2*A* and *SI Appendix*, Fig. S10). We first prepared libraries of RNAs designed to form short stems with randomized 3′-overhangs of different lengths (4 to 7 nt) and then subjected the RNA pools to the aminoacylation capture reaction. The ligated products were first purified, then treated to remove the amino acid (*Materials and Methods*) and finally enzymatically religated to generate an RNA library for suitable for reverse transcription, PCR amplification, and deep sequencing. The resulting distribution of reads, normalized to the distribution in the starting library, suggested that, under our reaction conditions, a small proportion of sequences confer highly efficient amino acid–bridged loop-closing ligation (*SI Appendix*, Fig. S11). Although synthesizing and assaying the sequences with the highest number of reads for 4, 5, and 6-nt overhangs did not result in the identification of particularly high-yielding sequences (Fig. 2*B*), we were gratified to observe that the 7-nt overhangs with the highest number of reads resulted in a near-quantitative yield of the loop-closed ligation product (Fig. 2*B*).

To understand why the specific 7-nt overhang led to such a high yield of loop-closed product, we first examined the kinetics of the loop-closing reaction as a function of time and concentration of activated glycine. The capture reaction using the 5′-UGAGAAA-3′ overhang reached ≥80% yield after 24 h even with only 1 mM glycine and CDI, which is 50-fold less than used in all earlier experiments (*SI Appendix*, Fig. S12*A*). The strong and saturable dependence of rate on substrate concentration suggested that the RNA overhang might fold into a structure that binds the activated glycine substrate. In addition, this construct failed to produce any loop-closed product with several other amino acids (*SI Appendix*, Fig. S12*B*), consistent with the formation of a defined substrate binding site. Interestingly, this overhang sequence produced minimal RNA-only ligation product in the absence of glycine, even with an increased Mg^2+^ concentration and the addition of 1-methylimidazole (*SI Appendix*, Fig. S12*C*), conditions known to afford up to 30 percent ligated RNA product (33) in the context of different overhang sequences. These results suggest that there may be distinct steric requirements for different activated amino acids, as well as for aminoacylation-dependent and -independent loop-closing ligations.

To gain insight into the sequence requirements for efficient loop-closing ligation, we examined our entire sequencing dataset more closely. The sequencing data for all four overhangs revealed a continuous enrichment in AU-content as a function of reads (*SI Appendix*, Fig. S13). An AU-rich sequence composition is known to be associated with higher flexibility and may facilitate bringing the overhang 3′-terminus close to the 5′-phosphorimidazolide of the capture strand (34, 35). All single mutations of the 5′-UGAGAAA-3′ overhang at positions 1, 2, 5, 6, and 7 corresponded to sequences that had a lower number of reads (Fig. 2*C*), suggesting that a highly sequence-specific architecture is needed to maximize capture efficiency. We searched for additional sequences that might yield efficient capture by training a booster tree regression model and obtaining estimated impacts of sequence features on the final read counts by employing SHAP analysis. Clustering the Shapley values using a K-means algorithm and synthesizing the second- and third-best cluster representatives yielded much poorer glycine capture (*SI Appendix*, Fig. S14), implying that sequences like 5′-UGAGAAA-3′ possess unique features necessary for efficient capture. To shed light on those features and the synergistic effects in the top cluster sequences, we built a median interaction matrix using the SHAP python package (Fig. 2*D*). Strong positive interactions between 1-U, 2-G, and 5-6-7-A define this top sequence cluster, strengthening the notion that this sequence forms a coordinated architecture that facilitates aminoacylation and ligation.

### Structural Elements Required for Aminoacylation-Dependent Ligation.

**Fig. 3.**
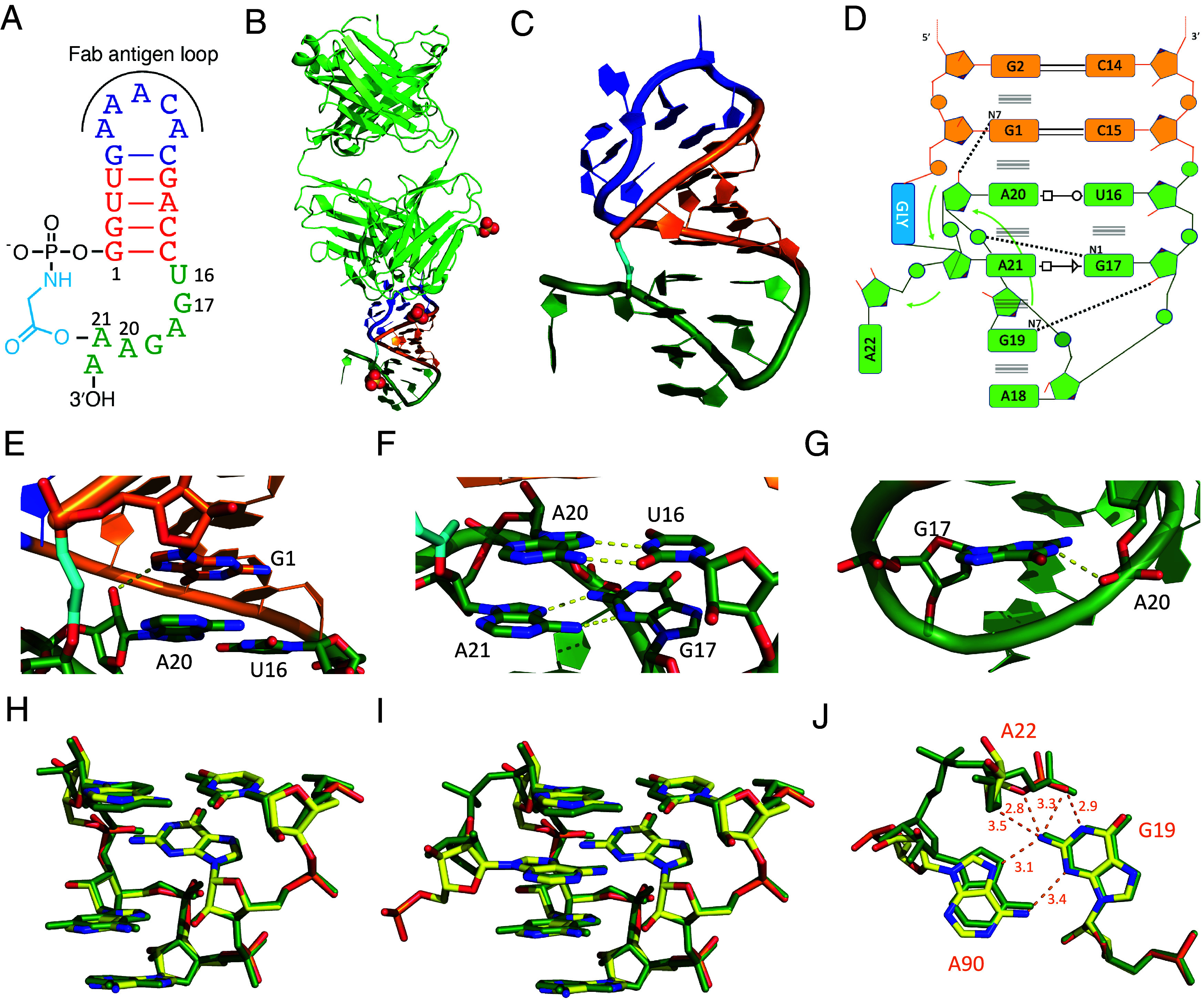
Secondary and tertiary structures of the glycine-bridged ligated loop construct as determined by X-ray crystallography. (*A*) Schematic secondary structure of the overall chimeric dumbbell RNA crystallization construct. (*B*) Overall arrangement of the ligated loop construct and Fab BL3-6 in the crystal structure, with the ligated loop in green, glycine linker in cyan, sulphate ions as spheres. (*C*) Tertiary structure of the ligated loop construct. (*D*) Secondary structure of the ligated loop; interactions are denoted using the Leontis–Westhof nomenclature. (*E*) A20:2′OH interacts with G1:N7. (*F*) Parallel base pairing interactions between A20 and U16 & A21 and G17 in the ligated loop. (*G*) G17:N1 interacts with A20:OP2. (*H*) Superposition of ligated loop structural motif (green) and the T-loop motif (yellow) from the FMN riboswitch (PDB 3F2T) without the intercalating base. (*I*) as in *H* but with the intercalating base. (*J*) as in *I*, showing distances and possible interactions between G19, A22, and A90 of the FMN riboswitch.

In an effort to understand the efficient glycine aminoacylation and loop-closing ligation mediated by the 5′-UGAG AAA-3′ sequence, we set out to determine the structure of the loop-closed product. To accomplish this, we designed an RNA construct that consists of two loops connected by a five base-pair duplex region (Fig. 3*A* and *SI Appendix*, Fig. S15*A*). One loop contains a sequence (5′-AAACA-3′) that binds to the antibody fragment Fab BL3-6 (36), and the other loop contains the aminoacylated overhang sequence (5′-UGAGAAA-3′). Following aminoacylation and loop-closing ligation, the dumbbell shaped product RNA was used to screen for optimal crystallization conditions. Crystals that diffracted well were obtained, and the structure of the RNA–Fab complex was solved to 2.07 Å resolution with an R_work_ of 22% and an R_free_ of 25% (PDB accession code 9AUS (37), *SI Appendix*, Table S2). The asymmetric unit contained two Fab–RNA complexes, one of which was more ordered than the other and is the subject of the analysis described below.

**Fig. 4.**
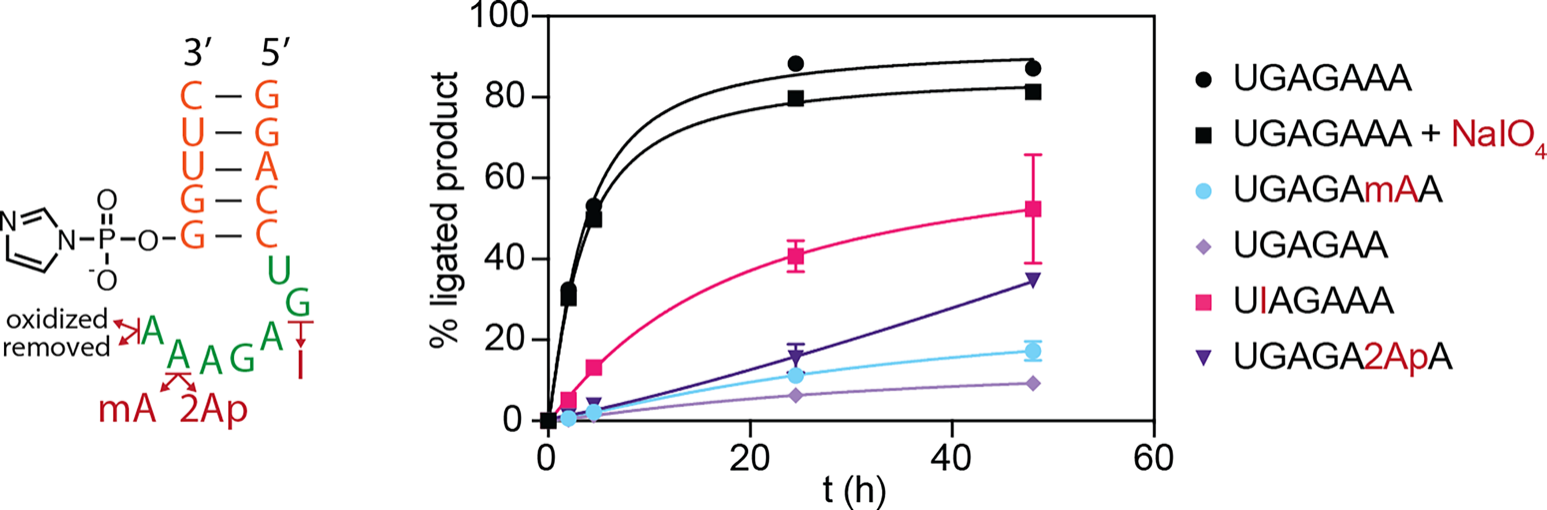
Effect of modifications of the overhang on the loop-closing reaction. The capture reaction was performed with the acceptor RNA modified as indicated. Loop-closing ligation reactions were performed in technical triplicates in 100 mM imidazole pH 8, 5 mM MgCl_2_, 1 µM oligonucleotides, and 50 mM CDI + gly at 0 °C. mA is 2′-OMe-A; I is inosine; 2Ap is 2-aminopurine. The hyperbolic fit lines serve to guide the eye and do not represent a kinetic fit.

Interpreting the electron density map was challenging at first, because the distance between the 5′-phosphate of the RNA and the 3′-hydroxyl was far too great to be consistent with the expected glycine bridge. To our surprise, iterative rounds of model building and refinement revealed positive density corresponding to the glycine linker between the 5′-end of the P1 stem (G1) and the 2′-OH of the penultimate residue of the 5′-UGAGAAA-3′ sequence (i.e., A21 of the crystallization construct) (*SI Appendix*, Fig. S16*A*). As a result, the 3′-terminal A22 of the overhang sequence protrudes from the structure of the closed dumbbell-shaped ligated RNA (Fig. 3*C*). This structure was highly unexpected, because of the demonstrated participation of the 2′,3′-diol in aminoacylation for a different, previously studied construct (*SI Appendix*, Fig. S7). The 5′-P to 2′-hydroxyl ligated product is a unique RNA branching reaction, mediated by glycine, which leaves the 3′-terminus available as a handle for subsequent binding events and reactions. We carried out several tests to confirm that the site of aminoacylation in the 5′-UGAGAAA-3′ sequence was indeed an internal 2′-hydroxyl and not the expected terminal 2′,3′-cis-diol of the RNA. First, oxidation of the cis-diol by periodate treatment had no effect on the aminoacylation-mediated loop-closing reaction (Fig. 4). In contrast, 2′-O-methylation of the penultimate A in the acceptor strand loop significantly inhibited the reaction. In addition, truncation of the overhang to 5′-UGAGAA-3′ had the same effect as 2′-O-methylation of the penultimate A, indicating that only the complete 7-nt overhang can readily adopt the conformation necessary for efficient aminoacylation-dependent loop closing (Figs. 3 and 4).

In the crystal structure of the glycine-bridged stem-loop, the 2′-aminoacylated loop adopts an architecture that is stacked on and continues the central double-stranded region of the dumbbell-shaped RNA. The folded overhang sequence 5′-UGAGAAA-3′ exhibits a number of striking features. The first two nucleotides of the 7-nt overhang, U16 and G17, form consecutive noncanonical base-pairing interactions with the Hoogsteen edges of downstream adenosine residues A20 and A21, continuing the P1 duplex stack and creating a stem-loop-like structure (Fig. 3 *C*–*F*). The string of stacked purines continues below A20 and A21 with G19 and A18, but these nucleotides are not stacked in consecutive order. Rather, the backbone linking G19 and A20 is fully stretched out, leaving a pocket in between these two residues. The backbone then makes an abrupt turn at A20, reversing direction and allowing A21 to be sandwiched into this pocket (Fig. 3*C*). The four nucleotides G17, A18, G19, and then the nonconsecutive A21 form the loop that caps the extended base-paired stem (Fig. 3 *C* and *D*). The overall structure of the folded and ligated overhang sequence exhibits a dense network of stacking and pairing interactions, supplemented by three additional hydrogen bonds that likely stabilize the observed configuration (Fig. 3 *E*–*G*). These interactions were evident in our deep sequencing data analysis (Fig. 2*D*), suggesting that future aminoacylation efforts with RNA architectures other than hairpin loops could rely on our computational pipeline to inform on potential RNA structures. Interestingly, residue A18, which makes no nucleobase-specific hydrogen-bonding interactions with other residues (but is stacked on G19), can be replaced with U or C with little effect on the yield of loop-closing ligation (Fig. 2 *B*–*D*).

Surprisingly, methylation of the site of aminoacylation, i.e., the 2′-hydroxyl of the penultimate A, only partially inhibited the loop-closing reaction. To determine the structure of the resulting alternative product, we scaled up the reaction and purified the loop-closed A21 2′-OMe RNA (*SI Appendix*, Fig. S15*B*). We then crystallized the RNA–Fab complex and determined its structure (PDB accession code 9AUR (38), *SI Appendix*, Table S2 and Figs. S16*B* and S17). In this structure, the glycine linker lies between the terminal 5′-phosphate (G1) and the 3′-terminal A22 ribose (*SI Appendix*, Fig. S17 *A*–*D*). However, we cannot resolve whether the aminoacyl group esterifies the 2′-OH or the 3′-OH or possibly a mixture of the two. Overall, the loop-closed 2′-OMe-modified overhang adopts a structure that is highly similar to that of the unmodified overhang sequence, except that the aminoacylated residue A22 now occupies the pocket previously occupied by the aminoacylated A21 (*SI Appendix*, Fig. S17 *E*–*J*). Conversely, the nonaminoacylated residue A21 protrudes from the folded structure into the solvent, as did the nonaminoacylated residue A22 of the original construct. The tertiary interactions stabilizing the closed-loop structure are the same in both structures, except for the backbone path flanking the aminoacylated residue (*SI Appendix*, Fig. S17 *D*–*J*).

Close examination of the backbone and nucleobase arrangements of the first five nucleotides of the overhang sequence (5′-UGAGA-3′) revealed that both structures bear a strong resemblance to the T-loop architecture (Fig. 3*H* and *SI Appendix*, Fig. S18*A*), a ubiquitous motif that frequently mediates tertiary interactions within noncoding RNAs, including the T-loop of tRNA (39–42). A subclass of these compact U-turn-like structures creates a pocket in which a nucleobase can intercalate between nucleobases 4 and 5, forming a continuous base-stack, and can also interact with nucleobase 2 through hydrogen bonding (Fig. 3 *I* and *J* and *SI Appendix*, Fig. S18 *B* and *C*) (43, 44). Indeed, within structured RNAs, T-loops can serve as receptors for adenosine residues forming a trans sugar edge/Hoogsteen interaction with a guanosine at position 2 (Fig. 3 *I* and *J*) (45, 46), analogous to the A21 or A22 insertions observed in our constructs.

Considering that nicked duplexes and other overhang sequences ligate much less efficiently than the nicked hairpin loop construct, the T-loop architecture observed in our structures may be responsible for facilitating ligation. To test the importance of the folded structure for capture efficiency, we prepared RNAs in which individual functional groups in the overhang were removed or altered. To examine the role of the sheared pair involving the intercalated A and G17, we prepared two constructs, one in which G17 was replaced with inosine, and one in which A21 was replaced by 2-aminopurine. Both constructs ligated much less efficiently than the original sequence, supporting the hypothesis that the observed tertiary interactions contribute to ligation efficiency (Fig. 4).

### Ribozyme Assembly by Iterated Loop-Closing Ligation.

**Fig. 5.**
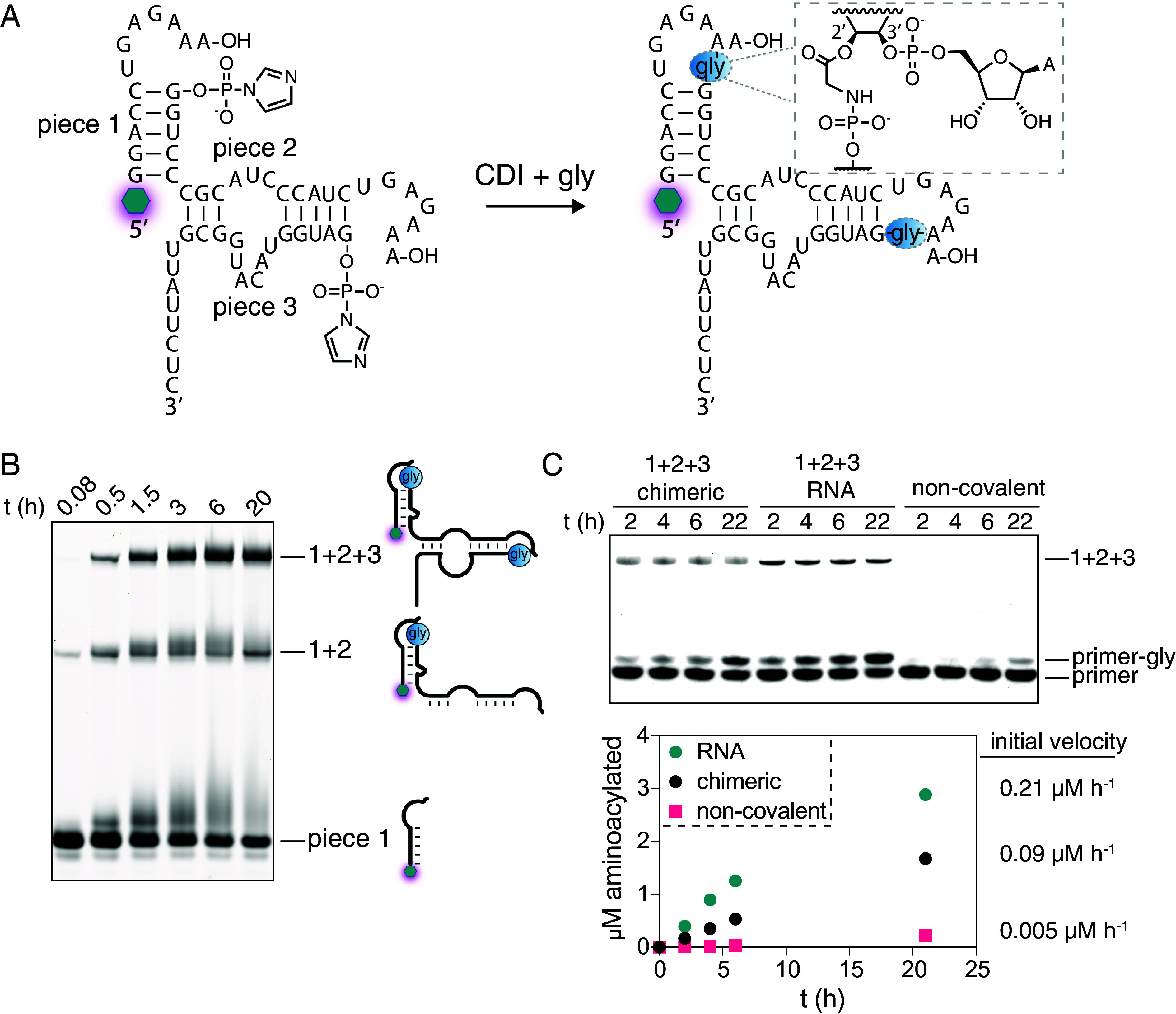
Template-free assembly of a chimeric Flexizyme by two loop-mediated activated glycine capture reactions. (*A*) Schematic and secondary structure of the modified Flexizyme ribozyme. The glowing hexagon represents 5′-fluorescein. The blue circles represent the glycine bridge, connecting the RNA as shown in the inset. (*B*) Denaturing PAGE of the activated glycine capture reaction over time. The reaction was performed in technical triplicates in 100 mM imidazole pH 8, 5 mM MgCl_2_, 5 µM each oligonucleotide, 50 mM CDI + gly at 0 °C. (*C*) *Top*: denaturing acidic PAGE of the aminoacylation reaction. *Bottom*: timecourse of the aminoacylation reaction quantified by measuring the ratio of the glycylated vs. not glycylated gel band in each lane. The reaction was performed in technical triplicates in 100 mM imidazole pH 8, 5 mM MgCl_2_, 5 µM primer oligonucleotide, 0.5 µM ribozyme, 5 mM DBE-gly (20 % overall DMSO) at 0 °C. The noncovalent ribozyme consisted of the complex assembled from the three unactivated component oligonucleotides.

The high yield of the glycine-mediated loop-closing reaction suggested a potential pathway for the structure-guided assembly of ribozymes from small RNA fragments (47–50). We have previously shown that functional chimeric ribozymes can be assembled via multiple template-directed ligations, but that strategy relies on presynthesized aminoacylated RNAs and some means of removing the splint templates so that the ribozyme sequence can fold (9). More recently, in a proof-of-principle study, we have shown that an RNA-only loop-closing ligation can be used to assemble hammerhead and ligase ribozymes from two oligonucleotide components (33). To investigate whether the glycine-mediated loop-closing ligation could be used to assemble ribozymes without an external template, we divided the dFx Flexizyme into three segments and placed 5′-UGAGAAA-3′ overhangs at each of the two duplex stems (Fig. 5*A*) (6, 51). Upon incubating the modified construct with 50 mM CDI-activated glycine, we observed rapid ligation that yielded the full-length chimeric ribozyme in 43 % yield (Fig. 5*B*). The aminoacylation activity of the isolated chimeric Flexizyme was about half that of the all-RNA ribozyme without amino acid bridges, but 18-fold higher than that of the noncovalently assembled ribozyme (Fig. 5*C*). The high activity of the chimeric Flexizyme suggests that the glycine bridges are well tolerated in the two T-loops, in contrast to their inhibitory effect when placed near the active site (9). The efficient aminoacylation-driven untemplated assembly of the Flexizyme points to a possible role for this type of process in the primordial assembly of ribozymes within primitive protocells in which RNA replication is still limited to short unstructured oligonucleotides, and larger structured ribozymes cannot easily be replicated.

## Discussion

Our work addresses the possibility that RNA aminoacylation served a primordial function that was advantageous at an early stage of the RNA World. Such a function would help to explain the evolutionary origins of the translation system. We have previously shown that RNA aminoacylation can lead to enhanced rates of template-directed RNA ligation, but achieving enhanced ligation yields required preaminoacylated RNAs, which we had to generate by ribozyme-catalyzed aminoacylation. The low steady-state levels of RNA aminoacylation that can be achieved by all known pathways for nonenzymatic aminoacylation have made it difficult to imagine how a biologically advantageous function could result from purely chemical aminoacylation. We have now found that specific RNA 3′-overhang sequences can be efficiently aminoacylated in the presence of amino acids that are activated as imidazolides. Attack of the amine of the amino acid on the activated 5′-phosphate of the complementary strand of the duplex, combined with aminoacyl ester formation, results in loop closing ligation and stabilization of the otherwise labile aminoacyl ester. Thus, the presence of both carboxyl and phosphate activating chemistry can lead to the formation of covalently closed hairpin loops with a bridging amino acid at the ligation junction. We have shown that this template-independent mode of ligation can be iterated to generate larger structured RNAs with ribozyme activity, including a ribozyme that is itself an aminoacyl-RNA synthetase. We suggest that similar processes may have acted within protocells at an early stage of the RNA World to enhance the assembly of ribozymes from sets of short oligonucleotides, such as those that might constitute a fragmented genome (52).

While the mechanism by which the 5′-UGAGAAA-3′ sequence facilitates the aminoacylation and loop-closing ligation reaction remains unclear, several arguments suggest that the unligated overhang sequence may be preorganized in a manner similar to the product structure and that this structure catalyzes one or both reactions. First, the site of aminoacylation was, to our surprise, the internal 2′-hydroxyl of the penultimate (−1 position) nucleotide of the overhang, and not the expected cis-diol of the 3′-terminal nucleotide. The −1 nucleotide is intercalated between two nucleotides of the T-loop structure, possibly positioning its 2′-hydroxyl in a manner favoring its aminoacylation and/or facilitating subsequent attack of the glycine amine on the nearby activated 5′-phosphate. In addition, the loop-closing ligation reaction is highly specific for glycine. Finally, destabilization of the folded structure by chemical mutagenesis greatly reduces the yield of loop-closing ligation. Thus, preorganization of the 3′-overhang sequence in the folded geometry provides potential explanations for both the site and amino acid specificity of the loop-closing reaction. This finding of internal 2′-OH aminoacylation reinforces the previous precedent that within structured RNA, the 3′-terminus may not be the most reactive site for aminoacylation (53). The crystal structure of the ligated product also provides a potential explanation for the increased stability of the aminoacyl ester linkage, because the folded structure restricts the solvent-accessible surface area of the ester by 90%, which would not occur in duplex or unstructured strands (*SI Appendix*, Fig. S19).

The chimeric glycine-bridged RNA folds into a stable compact structure with the same 3D architecture as seen in the canonical T-loop that is widely distributed in biological RNA structures, including the eponymous T-loop of tRNAs. This motif is so small and simple that it is likely to have evolved independently many times wherever it was needed to fulfill a structural or, as our results now suggest, a catalytic role. This raises the question of whether other, perhaps more complex and less widely distributed, structural motifs might also exist, some of which might also facilitate aminoacylation and ligation reactions. We are currently screening RNA libraries for internal loop and bulge-closing ligation reactions, using the same type of deep sequencing assay that allowed us to screen 3′-overhangs for enhanced loop-closing ligation. The identification of sequences that enable additional structure-generating ligation reactions would greatly increase the diversity of RNA structures that could be assembled as a result of aminoacylation and ligation. We are particularly interested in the identification of RNA sequences that confer different amino acid specificities on the aminoacylation reaction, as this could allow for the strategic positioning of bridging amino acids within a folded RNA such that the amino acid side chains could stabilize or modulate the folded structure or even enhance the catalytic abilities of an assembled ribozyme. While the self-assembly of such chimeric RNAs involves intrinsic sequence and amino acid specificity, these constraints might be reduced if the aminoacylation step was carried out by distinct ribozymes. Indeed, our assembly of a highly active chimeric Flexizyme variant using this chemistry highlights how aminoacyl-RNA esters that are captured as phosphoramidates can lead to a catalyst that efficiently synthesizes aminoacyl-RNAs that are bona fide ribosomal substrate, i.e., 2′(3′)-aminoacyl esters. We, therefore, suggest that the assembly of chimeric ribozymes with enhanced activities could result in a selective pressure for the evolution of ribozyme aminoacyl-RNA synthetases that are both sequence and amino acid specific, thus generating the substrates later coopted for the evolution of coded peptide synthesis.

## Materials and Methods

The standard aminoacyl-RNA capture reaction was performed at 0 °C and contained 10 or 1 µM of FAM-labeled acceptor oligonucleotide and capture oligonucleotide each, 5 mM MgCl_2_, 100 mM imidazole pH 8.0, and 50 mM or less of preactivated glycine. The reaction was analyzed by 20 % denaturing urea-PAGE, and the percent product was obtained by quantifying the per-lane normalized band intensity in ImageQuant TL software. Following the deep sequencing on the Illumina MiSeq, the data were analyzed using the code posted at https://github.com/szostaklab/aminoacylation/blob/main/Hydro-seq_example.ipynb. The sequencing data were additionally analyzed using the Python XGBoost package (54), the Python SHAP package (55), and the schemaball interaction network with a modified version of Oleg Komarov MATLAB code (56). The chimeric constructs for X-ray crystallography were incubated with 1.1 molar equivalents of Fab for 2 to 3 wk at 4 °C. Diffraction data were collected at APS beam line 24-ID-C, and the datasets were integrated and scaled using the on-site RAPD automated programs (https://rapd.nec.aps.anl.gov). The structures were solved using molecular replacement of the Fab BL3-6 from the previously reported structure [PDB code: 7SZU (57)] as search model in Phenix Phaser (58), and the RNA was built into the emerging density after multiple rounds of refinement using Coot and Phenix Refine (59–61). The modified, chimeric Flexizyme was synthesized by incubating 5 mM MgCl_2_, 100 mM imidazole pH 8.0, 5 µM FAM-labeled piece 1, 5 µM piece 2, 5 µM piece 3, and 50 mM preactivated glycine at 22 °C for 20 h. Following urea-PAGE purification, the chimeric Flexizyme activity was assessed by incubating 5 µM FAM-labeled RNA substrate, 0.5 µM Flexizyme, 100 mM imidazole pH 8.0, 5 mM MgCl_2_, and 5 mM DBE-gly (20 vol% DMSO) at 0 °C. For a detailed description of the methods, please see *SI Appendix*, Supporting Text.

## Supplementary Material

Appendix 01 (PDF)

## Data Availability

Structural and code data have been deposited in PDB (9AUS and 9AUR) (37, 38) and GitHub (62), respectively. All other data are included in the manuscript and/or *SI Appendix*.
