# Supplementary material for: A potential role for RNA aminoacylation prior to its role in peptide synthesis
Source: Proc Natl Acad Sci U S A. 2024 Aug 23;121(35):e2410206121. doi: 10.1073/pnas.2410206121 (PMC11363276; doi:10.1073/pnas.2410206121)
Supplement: Supplementary file 1 — Appendix 01 (PDF) [file pnas.2410206121.sapp.pdf]

PNAS

Supporting Information for

A potential role for RNA aminoacylation prior to its role in peptide synthesis

Aleksandar Radakovic[1,2], Anna Lewicka[3], Marco Todisco[1], Harry R. M. Aitken[4,5], Zoe Weiss[6], Shannon Kim[1], Abdullah Bannan[6], Joseph A. Piccirilli[1,3] & Jack W. Szostak[1 *]

Affiliations:

[1]Howard Hughes Medical Institute, Department of Chemistry, The University of Chicago; Chicago, Illinois 60637, USA.

[2]Department of Genetics, Harvard Medical School; Boston, Massachusetts 02115, USA.

[3]Department of Biochemistry and Molecular Biology, The University of Chicago; Chicago, IL 60637, USA.

[4]Howard Hughes Medical Institute, Department of Molecular Biology and Center for Computational and Integrative Biology, Massachusetts General Hospital; 185 Cambridge Street, Boston, Massachusetts 02114, USA.

[5]Department of Chemistry and Chemical Biology, Harvard University; Cambridge, Massachusetts 02138, USA.

[6]Department of Molecular and Cellular Biology, Harvard University; Cambridge, Massachusetts 02138, USA.

*Jack W. Szostak

Email: jwszostak@uchicago.edu

This PDF file includes:

Supporting text

SI References

Scheme S1

Figures S1 to S21

Tables S1 to S3

Supporting text

Safety Information.

No unexpected or unusually high safety hazards were encountered.

General information.

All reagents were purchased from Sigma-Aldrich and Fisher Scientific, unless otherwise noted. Oligonucleotide synthesis reagents were purchased from ChemGenes and Glen Research. Illumina sequencing reagents were purchased from Illumina and New England Biolabs.

Common protocols and recipes.

Ethanol precipitation: 0.1 volumes of 3 M sodium acetate pH 5.5 and at least 3 volumes of ethanol were added to the RNA solution. The precipitation was allowed to proceed for 20 minutes on dry ice before being centrifuged at maximum speed at 4 ºC. The supernatant was removed, and the pellet was washed twice with cold 80 % ethanol.

Isopropanol precipitation: 0.1 volumes of 5 M ammonium acetate and at least 3 volumes of isopropanol were added to the RNA solution. The precipitation was allowed to proceed for 20 minutes on dry ice before being centrifuged at maximum speed at 4 ºC. The supernatant was removed, and the pellet was washed twice with cold 80 % ethanol.

Soaking buffer: 5 mM sodium acetate pH 5.5 mixed with 2 mM EDTA pH 8.0. The measured pH of this solution was 7 without adjustments.

Acidic soaking buffer: 5 mM sodium acetate pH 5.5 mixed with 2 mM EDTA pH 8.0. The pH was adjusted to 5.0 with 1 M HCl.

Quenching buffer: 50 mM EDTA pH 8.0, 1x bromophenol blue, 95 % v/v formamide.

Acidic quenching buffer: 10 mM EDTA pH 8.0, 1x bromophenol blue, 100 mM sodium acetate pH 5.0, 150 mM HCl, 75 % v/v formamide.

Oligonucleotide synthesis.

All oligonucleotides were synthesized on the K&A H-6 solid-phase DNA/RNA synthesizer. Randomized nucleotides in the sequencing constructs were introduced from a hand-mixed solution containing 3:3:2:2 A:C:G:U phosphoramidites. Following cleavage and deprotection as recommended by the manufacturer, the oligonucleotides were evaporated in a speed-vac for 2 hours and lyophilized for 16 hours. Removal of the 2′-O-TBDMS groups was performed in 100 µL DMSO and 125 µL triethylamine trihydrofluoride at 65 ºC for 2.5 hours. Oligonucleotides were then precipitated with isopropanol, dissolved in 5 mM EDTA in 99

% v/v formamide, and purified by denaturing urea-PAGE. The desired gel bands were cut, crushed up, and soaked in soaking buffer for 16 hours. Finally, the oligonucleotides were filtered and desalted with either C18 Sep-Pak cartridges (Waters) for lengths <25 nt or Amicon MWCO filters for lengths >25 nt. Analysis of pure RNA was performed on an Agilent 6540 mass spectrometer.

Oligonucleotide activation.

To a solution of 250 µM RNA in 125 mM imidazole pH 7 was added EDC.HCl, so that the final 300 µL solution contained 200 µM RNA, 100 mM imidazole pH 7, and 100 mM EDC.HCl. The reaction was rotated for 2 hours at 22 ºC before being precipitated with 30 µL acetone saturated with sodium perchlorate and 1 mL acetone on dry ice for 10 minutes. After centrifugation and supernatant removal, the pellet was washed twice with a 1:1 v/v mixture of acetone and diethyl ether. Activation efficiency was determined by HPLC using the Atlantis TM T3 column (3 µm, 4.6 x 150 mm) at a flow rate of 0.5 mL/min. The following gradient was used: (A) aqueous 50 mM triethylammonium acetate (pH 7.0) and (B) acetonitrile, from 6% to 12% B over 20 min. Activation efficiency was routinely over 85 %.

Activation with 2-methylimidazole was performed as described previously(1).

Model monomer aminoacylation (Figure S1).

Model MeO-pA was synthesized following the reported procedure of Ivanovskaya and coworkers(2). Aminoacylation was attempted using either (i) CDI + glycine or (ii) gly-NCA(3) + imidazole, in D_2O at pD = 7.5 (imidazole buffer). Experiments were incubated and monitored using time course ^1H NMR, sampling every 10 min for 4 h.

A: To a solution of glycine (94 mg, 1.25 M, 50 eq.) in D_2O (1 mL) was added carbonyldiimidazole (244 mg, 1.5 M, 60 eq.) and the mixture was rapidly stirred for 2 min. Next, 100 µL of the aforementioned solution was transferred to a solution of MeO-pA (6.25 mM) in imidazole buffer (pD = 7.5, 625 mM in D_2O, 400 µL). The combined mixture was vortexed for 30 s before being transferred to an NMR tube. The progress of the reaction was monitored by ^1H NMR spectroscopy over the course of 4 h.

B: To a solution of MeO-pA (6.25 mM) in imidazole buffer (pD = 7.5, 625 mM in D_2O, 400 µL) was slowly added a solution of gly-NCA (1.25 M in d 6 -DMSO, 100 µL, 50 eq.) with vigorous stirring. The combined mixture was vortexed for 1 min before being transferred to an NMR tube. The progress of the reaction was monitored by ^1H NMR spectroscopy over the course of 4 h.

C: An authentic sample of 2′/3′-glycyl product for ^1H NMR comparison was prepared following the procedure of Sutherland and coworkers(4).

Glycine-NCA was prepared using the procedure reported by Akssira and co-workers(3): To a solution of N-Boc glycine (175 mg, 1.00 mmol) in methylene chloride (5 mL) at 0 ºC, was added phosphorus trichloride (87 µL, 1.20 mmol). The reaction mixture was stirred for 2 h then concentrated under reduced pressure and the residue washed with chloroform (2 x 10 mL), affording Glycine-NCA in a ~3:1 mixture with Glycine.HCl / Glycine.$PO(OH)_3$ (see Figure S20 for ^1H NMR spectrum), which was used without further purification.

Model oligonucleotide aminoacylation (Figure S2).

100 µL of a 0.5 M glycine solution in water were added to 9.75 mg (1.2 eq) of CDI and rapidly vortexed for 20 seconds. This solution was then added to a solution of RNA such that the final 50 µL reaction contained 5 µM RNA, 360 mM buffer, 40 mM $MgCl_2$, and 100 mM premixed gly + CDI. 1 µl aliquots were taken at the indicated time points, quenched with 9 µL acidic quench buffer and analyzed by acidic 20 % denaturing urea-PAGE. Acidic PAGE was made with 100 mM sodium acetate pH 5.0 instead of the usual 1x Tris-

Borate-EDTA, and it was run in this same buffer at 25 W for 2.5 hours at 4 ºC. The remaining reaction was precipitated with ethanol, desalted with a zip-tip C18 column, and injected into the Agilent 6540 mass spectrometer.

Flexizyme-catalyzed oligonucleotide aminoacylation (Figures S2, S4, and 5C).

To a solution of RNA and Flexizyme dFx(5) or the modified dFx_S7 (Table S3) in imidazole pH 8.0 and $MgCl_2$ was added dinitrobenzyl ester of glycine (DBE-gly) so that the final concentrations were 5 µM RNA, 0.5 µM Flexizyme, 100 mM imidazole pH 8.0, 5 mM $MgCl_2$, and 5 mM DBE-gly (20 vol% DMSO). Aminoacylation efficiency was analyzed as above.

Amino acid capture reaction (Figures 1, S3, S5-9, S12, S14).

The following standard conditions were used unless otherwise noted: to a solution of 5.55 mM $MgCl_2$, 111 mM imidazole pH 8.0, 11.1 µM acceptor oligonucleotide, 11.1 µM capture oligonucleotide in 18 µL was added 500 mM of premixed gly + CDI in 0.5 µL portions every 24 hours over 96 hours at 0 ºC. The reaction was quenched every 24 hours by mixing 0.5 µL aliquots with 4.5 µL of quenching buffer and analyzed by 20 % denaturing urea-PAGE. The percent product was obtained by quantifying the per-lane normalized band intensity in the ImageQuant TL software.

The NaOH treatment shown in Figure S7B was performed by treating 4 µL of the quenched 96 hour timepoint with 1 µL of 1 M NaOH for 3 minutes at 22 ºC. The hydrolysis was quenched by the addition of 1 µL of 1 M HCl.

For the LC-TOF analysis in Figure S7C, a 25x scale reaction run for 96 hours was concentrated with Amicon MWCO filters, precipitated with ethanol, desalted with a zip-tip C18 column, and injected into the Agilent 6540 mass spectrometer.

Hydrolysis reaction (Table S1, Figure S8).

Synthesis: 500 µL reaction containing 5 mM $MgCl_2$, 100 mM imidazole pH 8.0, 10 µM acceptor oligonucleotide, 10 µM capture oligonucleotide, and 50 mM premixed gly + CDI was incubated for 96 hours at 0 ºC. The reaction was concentrated with Amicon MWCO filters to 50 µL, diluted with 50 µL formamide, and purified by 20 % denaturing urea-PAGE at 4 ºC. The desired product band was isolated, crushed, and soaked in acidic soaking buffer for 3 hours at 4 ºC. The solution was then filtered, concentrated with Amicon MWCO filters to 50 µL, and precipitated with ethanol.

Hydrolysis: 10 µL reactions containing 2.5 µM of the amino acid-bridged construct for the loop-closing reaction and an additional 50 µM duplexing oligonucleotide (A2C2duplex or A3C3duplex Table S3) for the nicked duplex reaction were heated at 90 ºC for 1 minute followed by slow cooling (0.1 ºC/s) to 22 ºC. The annealed reactions were then diluted with HEPES pH 8.0 and $MgCl_2$ to give the final conditions in 10 µL: 200 mM HEPES pH 8, 2.5 mM $MgCl_2$, 0.75 µM annealed construct. At the indicated time points, 1 µL aliquot was diluted with 14 µL quenching buffer containing 2.4 µM of the reverse complement of the duplexing oligonucleotide (A2C2revcomp or A3C3revcomp in Table S3), heated at 95 ºC for 3 minutes, quickly cooled on ice, and analyzed by 16 % denaturing urea-PAGE. The ratio of the full-length glycine-bridged construct to the hydrolyzed product was obtained by quantifying the per-lane normalized band intensities in ImageQuant TL. Negative natural logarithm of the ratio of the remaining bridged construct (P) to the initial bridged construct (P_0; assumed to be 1) at each time point was plotted against time. The slope represented the k_{obs}, and the half-lives were obtained by dividing ln(2) with the k_{obs}.

Deep sequencing (Figures 2, S10, S11).

Preparative reaction:

1. To a solution of 7.5 mM $MgCl_2$, 150 mM imidazole pH 8.0, 1.5 µM each acceptor oligonucleotide construct, 1.5 µM capture oligonucleotide per acceptor construct, 1.5 µM protecting oligonucleotide per construct in 2.666 mL was added 150 mM of premixed gly + CDI in 334 µL portions at 0 and 24 hours at 0 ºC. At 48 hours, the reaction was concentrated using Amicon MWCO filters to 50 µL, diluted with 50 µL formamide, and purified by 16 % denaturing urea-PAGE at 4 ºC. The product band was crushed, soaked in acidic soaking buffer for 3 hours, filtered, concentrated with Amicon MWCO filters, and desalted using the Zymo Oligo Clean and Concentrator kit.
2. The isolated product was then hydrolyzed in 200 mM NaOH for 3 minutes, quenched with HCl, diluted with formamide, purified by 16 % denaturing urea-PAGE, and isolated as above.
3. The hydrolyzed product was then ligated to the preadenylated RT primer binding oligonucleotide using the standard NEB protocol for T4 RNA ligase 2, truncated KQ with 10 % PEG at 25 ºC for 22 hours. To a 20 µL ligation reaction was added 0.5 µL Proteinase K and the reaction was incubated for 15 minutes at 22 ºC. The reaction was cleaned up with 2 washes of 25:24:1 v/v/v phenol:chloroform:isoamyl alcohol followed by desalting with the Zymo Oligo Clean and Concentrator kit.

At this step, the initial library that did not undergo steps 1 and 2 was also subjected to steps 3-7. This was done to capture the initial library sequence bias and correct for it by performing a normalization of the sequences that captured glycine.

4. The ligated product was reverse transcribed using the NEB ProtoScript® II First Strand cDNA Synthesis Kit and the cDNA was isolated using the Zymo Oligo Clean and Concentrator kit with the recommended alkaline RNA removal.
5. The cDNA (200 ng) was then PCR amplified and multiplexed with the NEB Q5 polymerase kit and NEBNext® Multiplex Oligos for Illumina® (Index Primers Set 1) using the standard Q5 PCR conditions for 6 cycles.
6. The amplified product was purified by 1.4 % agarose gel and extracted with the Monarch® PCR & DNA Cleanup Kit.
7. Prior to pooling, the final concentration of each sample was determined with the 4200 Agilent Tapestation. The samples were then pooled to the final total concentration of 1 nM and prepared for NGS per the Illumina protocol for Miseq, with a 30 % PhiX spike-in.

At steps 1, 2, and 3, 1 µL aliquots were diluted with quenching buffer and analyzed by 16 % denaturing urea-PAGE as in Figure S10.

Analytical reactions with individual sequences: the reactions were performed exactly as in step 1 above, except the reaction was scaled down 100x. Instead of continuing with the sequencing workflow, 1 µL aliquots were quenched at 48 hours in 9 µL quenching buffer and analyzed by 20 % denaturing urea-PAGE. The percent product was obtained by quantifying the per-lane normalized band intensity in the ImageQuant TL software.

General deep sequencing data analysis (Figures 2, S11).

The detailed and annotated custom python code for each construct analysis can be found at the following github link: https://github.com/szostaklab/aminoacylation/blob/main/Hydro-seq_example.ipynb.

The raw reads were first filtered for quality, the length of the randomized region, and the homemade barcode where applicable. The reads were then trimmed to only display the randomized regions of interest. The reads of the glycine capture reaction were then normalized as follows: for each unique sequence, we obtained the normalization factor by dividing the number of reads in the initial, control library by its expected number of reads if the library was truly random; then, we divided each unique sequence that came out of

the glycine capture reaction by its computed normalization factor. For example, if a sequence had 200 reads in the control library but we expected to see 100 reads, the normalization factor for that sequence would be 2. That same sequence in the glycine capture reaction would then be divided by 2, to account for its overrepresentation in the starting library. Sequences with less than 5 reads in the initial library were omitted from the analysis and assigned the read count of 0. This normalization procedure removes biases that occurred during the initial random library synthesis. The sequences and their corresponding read counts were then plotted and exported as excel files.

In-depth deep sequencing data analysis (Figures 2, S11, S13-14).

Most of the entries in our datasets corresponded to poorly represented sequences that we interpret as weakly reacting. For example, the average number of reads per unique sequence in our sequencing dataset for the 7-nt overhangs was only 20 reads, with a small proportion of sequences having >100 reads (see Figure S11). We were particularly interested in the sequences with the high number of reads and on the features that differentiate these sequences from the bulk.

The original sequencing dataset for 7 nt-long loops included 15765 sequences out of the 16384 possible heptamers formed by the canonical 4 nucleobases of RNA. The missing sequences were treated as zero-reads sequences, so that they are reintroduced in our expanded dataset to be taken into account for downstream analysis.

To extract quantitative features from the deep sequencing of the 7-nt overhangs, we trained a boosted tree regression model using Python XGBoost package(6). The dataset was split in training/test sets (80/20 split) and the sequences were One-Hot-Encoded to feed the model, so that every one of our 7-nt sequences resulted in a 7 x 4 matrix that was further flattened into a 28 elements-long array. The XGBoost model was trained with {max depth: 6, learning_rate: 0.1, subsample: 0.5, tree_method: 'hist'} and using our test set for early stopping (200 early stopping rounds) to reduce overfitting. Final RMSE for training set and test set were respectively equal to 7.9 and 12.5. The Shapley values were extracted from the trained model using Python SHAP package(7).

Before performing clustering on the Shapley values, we decided to modify the dataset by setting every Shapley value for zero-categorical features as equal to 0. Since most of the Shapley value variance belongs to categorical features equal to one (90% of Shapley values for zero-categorical features are comprised between -2.8 and 1.3, while 90% of Shapley values for one-categorical values are comprised between -3.9 and 8.3), we retain most of the model predictive power. Moreover, since zero-categorical features on average lower the read values predicted from the SHAP analysis, this effect can be partially compensated by assigning a net penalty of $\approx$10 to our reads. By discarding the zero-categorical features Shapley values, we ended up trading some performance of the model, with overall RMSE increasing from 10.0 to 14.9 (without net subtraction) or 11.1 (with net subtraction), while allowing for better interpretable clusters.

Clustering was performed using the K-means algorithm implemented in scikit-learn(8), with the optimal number of clusters chosen using the elbow method(9) as equal to 8.

To prepare data for visualization and ease the visualization of clusters with widely uneven size, we subsampled the modified Shapley dataset to contain no more than 300 entries per cluster. To generate the low-dimensional visualization of this reduced Shapley values dataset, we applied the UMAP algorithm implemented in Python UMAP package with neighbor number set to 150.

Pairwise interactions between sequence features were extracted using the SHAP package, and the resulting interaction network was visualized as a schemaball with a modified version of Oleg Komarov MATLAB code(10).

Amino acid capture with optimized UGAGAAA overhang sequence (Figures 3, 4, S12).

The loop-closing amino acid capture reaction, incubated at 0 ºC, contained the following components in 20 µL: 5 mM $MgCl_2$, 100 mM imidazole pH 8.0, 1 µM acceptor oligonucleotide, 1 µM capture oligonucleotide, and a range of concentrations between 50 mM and 1 mM of premixed amino acid and CDI.

Periodate oxidation (Figures 4, S7).

10 µM of acceptor oligonucleotide was treated with 10 mM sodium periodate in 200 µL for 1 hour at 0 ºC. The oligonucleotide was desalted using Amicon MWCO filters. The 50 µL solution was precipitated with ethanol.

Synthesis of the amino acid bridged constructs for X-ray crystallography (Figure S15).

Dumbell RNA (Figure 3): 80 µM acceptor A56, 80 µM capture C9, 25 mM EDTA, 100 mM imidazole pH 8, and 12.5 mM of premixed gly + CDI were mixed to a final volume of 8 mL and incubated for 48 hours at 0 ºC. The reaction was then concentrated using Amicon MWCO filters to 150 µL, diluted with 150 µL formamide, and purified using 20 % denaturing urea-PAGE. The desired amino acid-bridged product gel band was crushed, soaked in acidic soaking buffer for 3 hours at 4 ºC, filtered, desalted with C18 Sep-Pak cartridges, and lyophilized. The lyophilized product was then circularized to complete the Fab-binding loop using the NEB T4 RNA Ligase 1 (ssRNA Ligase), High Concentration under standard conditions for 60 minutes at the final reaction volume of 30 mL. The ligation reaction was stopped with 240 µL of Proteinase K for 15 minutes. The RNA solution was extracted twice 25:24:1 v/v/v phenol:chloroform:isoamyl alcohol followed by desalting with the C18 Sep-Pak cartridges. After lyophilization, the RNA was dissolved in 200 µL water and further desalted by precipitation with isopropanol.

The A21 2′-OMe dumbbell construct (Figure S16): 80 µM acceptor A57, 25 mM EDTA, 100 mM imidazole pH 8, and 12.5 mM of premixed gly + CDI were mixed to a final volume of 8 mL and incubated for 48 hours at 0 ºC. The reaction was then concentrated using Amicon MWCO filters to 150 µL, diluted with 150 µL formamide, and purified using 20 % denaturing urea-PAGE. The desired amino acid-bridged product gel band was crushed, soaked in acidic soaking buffer for 3 hours at 4 ºC, filtered, desalted with C18 Sep-Pak cartridges, and lyophilized.

Note: the circularized, amino acid-bridged product migrates faster than the corresponding linear amino acid-bridged RNA on the 20 % denaturing urea-PAGE.

Fab Purification.

The BL3-6 Fab expression vector (available upon request via an MTA; see Figure S21 for vector details) was transformed into 55244 chemically competent cells (www.atcc.org) and grown on LB plates supplemented with carbenicillin at 100 µg mL^{-1}. Nine colonies from the plate were chosen and inoculated to a starter culture with 100 µg mL^{-1} carbenicillin, which was grown at 30 °C for 8 hours. Once the starter culture reached an OD 600, 15 mL of starter culture was used to inoculate 1 L of 2×YT media and grown for 24 h at 30 °C. The cells were then pelleted via centrifugation at RT, and the cell pellet was resuspended in 1 L of freshly prepared CRAP-Pi media supplemented with 100 ug mL^{-1} carbenicillin. The cells were set to grow for 24 h at 30 °C, harvested via centrifugation at 4 °C and frozen at -20 °C. Frozen cell pellets were lysed in PBS supplemented with 0.4 mg mL^{-1} of Lysozyme and 0.01 mg mL^{-1} of DNase I. After 30 minutes PMSF was added to a final concentration of 0.5 mM. After 30 minutes, the mixture containing cellular debris and lysate was centrifuged, 45 min, 12000 rpm, rotor type JLA 16.250 (Beckman) at 4 °C. Lysate was transferred to new sterile bottles and centrifuged again for 15 minutes, 12000 rpm, at 4 °C. Supernatant was filtered through 0.45 µm filters into a sterile bottle (Millipore Sigma, www.sigmaaldrich.com), and Fab proteins were purified using the AKTAxpress fast protein liquid chromatography (FPLC) purification system (Amersham, www.gelifesciences.com) as described previously(11, 12). The lysate in PBS (pH 7.4) was

loaded into a protein A column, and the eluted Fab in 0.5 M acetic acid was buffer exchanged back into the PBS (pH 7.4) using 30 kDa cutoff Amicon filter and loaded into a protein G column. The Fab was eluted from protein G column in 0.1 M glycine (pH 2.7) and then buffer-exchanged into 50 mM NaOAc, 50 mM NaCl buffer (pH 5.5) and loaded into a heparin column. Finally, the eluted Fab in 50 mM NaOAc, 2 M NaCl (pH 5.5) was dialyzed back into 1× PBS (pH 7.4), concentrated, and analyzed by 12% SDS-PAGE using Coomassie Blue R-250 staining for visualization. Aliquots of Fab samples were tested for RNase activity using the RNaseAlert kit (Ambion, www.thermofisher.com). The aliquots of Fab samples were flash frozen in liquid nitrogen and stored at −80 °C until further use.

Crystallization.

The crystallization construct was analyzed by electrophoretic mobility shift assay under non-denaturing conditions. In the presence of a stoichiometric amount of Fab chaperone, BL3-6 RNA construct shifted quantitatively to the bound state. The same shift did not occur for a non-Fab sample. Validated RNA construct in ultrapure H_2O was subjected to crystallization with no further procedures of refolding. 480 µg of the RNA was mixed with a 1.1 molar equivalents of Fab BL3-6. The complex was then concentrated to 6 mg/mL final concentration of RNA (80 µL). 100 nL + 100 nL hanging drop crystal trials were set in commercially available crystallization kits from Hampton Research and Jena Bioscience using the Mosquito liquid handling robot (SPT Labtech) and allowed to grow for two to three weeks at 4 °C. First crystals grew in 3 days. Loop-closed dumbbell RNA crystallized in Peg Ion: 0.2 M Lithium sulfate monohydrate; 20% w/v Polyethylene glycol 3,350, pH 5.7. A21 2′-OMe dumbbell construct also crystallized in Peg Ion: 0.07 M Citric Acid; 0.03 BIS-TRIS Propane; 16% w/v Polyethylene glycol 3,350, pH 3.8. Crystals were looped and frozen in liquid nitrogen.

Crystallographic data processing (Figures S16, S19).

Diffraction data was collected at APS beam line 24-ID-C. Data sets were integrated and scaled using the on-site RAPD automated programs (https://rapd.nec.aps.anl.gov). The structures were solved using molecular replacement of the Fab BL3-6 from the previously reported structure (PDB code: 7SZU(13)) as search model in Phenix Phaser(14). Two copies of the RNA and Fab were discovered in the P 1 space group for the loop-closed dumbbell RNA. Only one copy of the A21 2′-OMe dumbbell construct RNA and the Fab was discovered in the unit cell in the P 1 21 1 space group. Using the initial phases from the molecular replacement solution, the RNA was able to be built into the emerging density after multiple rounds of refinement using Coot and Phenix Refine(15–17). Visualization and solvent accessible surface area (SASA) evaluation were conducted with PyMOL (The PyMOL Molecular Graphics System, Schrödinger, LLC).

Ribozyme assembly (Figure 5A,B).

The ribozyme assembly reaction, incubated at 22 ºC, contained the following components in 20 µL: 5 mM $MgCl_2$, 100 mM imidazole pH 8.0, 5 µM piece 1, 5 µM piece 2, 5 µM piece 3, and 50 mM premixed gly + CDI. At each time point, 1 µL aliquot was diluted in 9 µL quenching buffer and analyzed by 16 % denaturing urea-PAGE. The percent product was obtained by quantifying the per-lane normalized band intensities in ImageQuant TL.

References

1. A. Radakovic, T. H. Wright, V. S. Lelyveld, J. W. Szostak, A Potential Role for Aminoacylation in Primordial RNA Copying Chemistry. *Biochemistry* **60**, 477–488 (2021).

2. M. G. Ivanovskaya, M. B. Gottikh, Z. A. Shabarova, Modification of Oligo (Poly) Nucleotide Phosphomonoester Groups in Aqueous Solutions. *Nucleosides, Nucleotides & Nucleic Acids* **6**, 913–934 (1987).

3. M. Akssira, M. Boumzebra, H. Kasmi, A. Dahdouh, M. L. Roumestant, P. Viallefont, New routes to 1,4-benzodiazepin-2,5-diones. *Tetrahedron* **50**, 9051–9060 (1994).

4. L.-F. Wu, M. Su, Z. Liu, S. J. Bjork, J. D. Sutherland, Interstrand Aminoacyl Transfer in a tRNA Acceptor Stem-Overhang Mimic. *J Am Chem Soc* **143**, 11836–11842 (2021).

5. H. Murakami, A. Ohta, H. Ashigai, H. Suga, A highly flexible tRNA acylation method for non-natural polypeptide synthesis. *Nat Methods* **3**, 357–359 (2006).

6. T. Chen, C. Guestrin, XGBoost: A scalable tree boosting system. *Proceedings of the ACM SIGKDD International Conference on Knowledge Discovery and Data Mining* 785–794 (2016). https://doi.org/10.1145/2939672.2939785.

7. S. M. Lundberg, P. G. Allen, S.-I. Lee, A Unified Approach to Interpreting Model Predictions. https://doi.org/10.5555/3295222.3295230.

8. F. Pedregosa, *et al.*, Scikit-learn: Machine Learning in Python. *Journal of Machine Learning Research* **12**, 2825–2830 (2011).

9. V. Satopää, J. Albrecht, D. Irwin, B. Raghavan, Finding a “kneedle” in a haystack: Detecting knee points in system behavior. *Proceedings - International Conference on Distributed Computing Systems* 166–171 (2011). https://doi.org/10.1109/ICDCSW.2011.20.

10. GitHub - okomarov/schemaball: Matlab function to create schemaball plots. Available at: https://github.com/okomarov/schemaball [Accessed 22 December 2023].

11. Y. Koldobskaya, *et al.*, A portable RNA sequence whose recognition by a synthetic antibody facilitates structural determination. *Nature Structural & Molecular Biology 2010 18:1* **18**, 100–106 (2010).

12. J. D. Ye, *et al.*, Synthetic antibodies for specific recognition and crystallization of structured RNA. *Proceedings of the National Academy of Sciences of the United States of America* **105**, 82–87 (2008).

13. H. C. Rees, W. Gogacz, N. S. Li, D. Koirala, J. A. Piccirilli, Structural Basis for Fluorescence Activation by Pepper RNA. *ACS Chem Biol* **17**, 1866–1875 (2022).

14. P. D. Adams, *et al.*, PHENIX: a comprehensive Python-based system for macromolecular structure solution. *Acta Crystallogr D Biol Crystallogr* **66**, 213–221 (2010).

15. P. V Afonine, *et al.*, Towards automated crystallographic structure refinement with phenix. refine. *Acta Crystallogr D Biol Crystallogr* **68**, 352–367 (2012).

16. P. Emsley, B. Lohkamp, W. G. Scott, K. Cowtan, Features and development of Coot. *Acta Crystallogr D Biol Crystallogr* **66**, 486–501 (2010).

17. C. J. Williams, *et al.*, MolProbity: More and better reference data for improved all-atom structure validation. *Protein Science* **27**, 293–315 (2018).

18. Y. Otake, H. Nakamura, S. Fuse, Rapid and Mild Synthesis of Amino Acid N-Carboxy Anhydrides: Basic-to-Acidic Flash Switching in a Microflow Reactor. Angew. Chemie Int. Ed. 57, 11389–11393 (2018).

19. J. Fan, J. Zou, X. He, F. Zhang, S. Zhang, J. E. Raymond, K. L. Wooley, Tunable mechano-responsive organogels by ring-opening copolymerizations of N -carboxyanhydrides. Chem. Sci. 5, 141–150 (2013).

Scheme S1: CDI-mediated glycine activation generates both NCAs and aminoacyl imidazolides. Shown are two possible paths (red and black) to NCA from the aminoacyl urea imidazolide intermediate (ref. *32* main text).

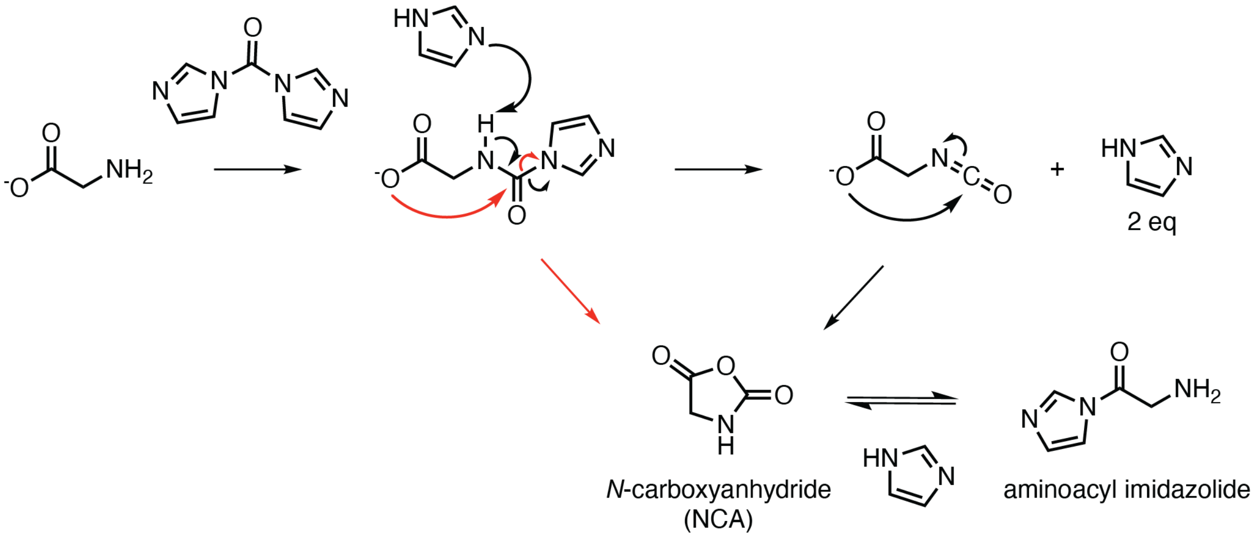

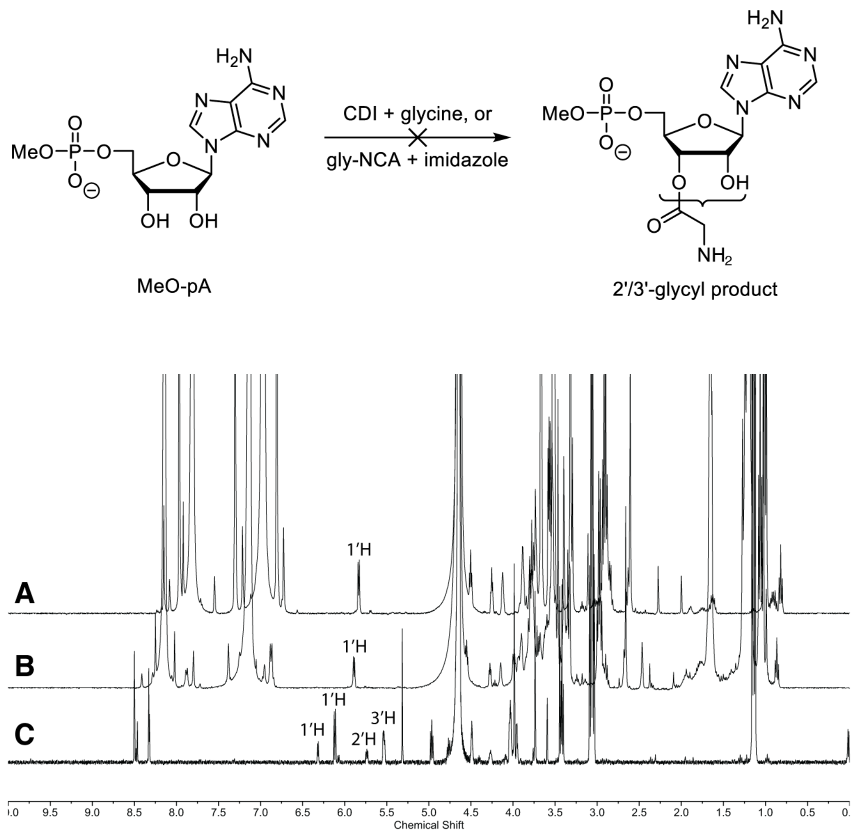

Figure S1. Aminoacylation of a model monomer, adenosine 5′-(O-methylphosphate). Representative ^{1}H NMR after 2 h of attempted aminoacylation of monomeric nucleotide (MeO-pA). **A:** No aminoacylation was observed in the reaction with premixed CDI + glycine; **B:** No aminoacylation was observed in the reaction with gly-NCA + imidazole; **C:** authentic product (2′/3′-glycyl-5′-MeO-pA). The region of the spectrum between 5 and 6.75 ppm contains the diagnostic signals of successful aminoacylation: 1′H is shifted downfield and appears as two signals due to the 2:1 equilibrium ratio of 3′-glycyl and 2′-glycyl ester. 2′H and 3′H are shifted downfield in the presence of glycyl ester and they also reflect the 2:1 equilibrium.

A

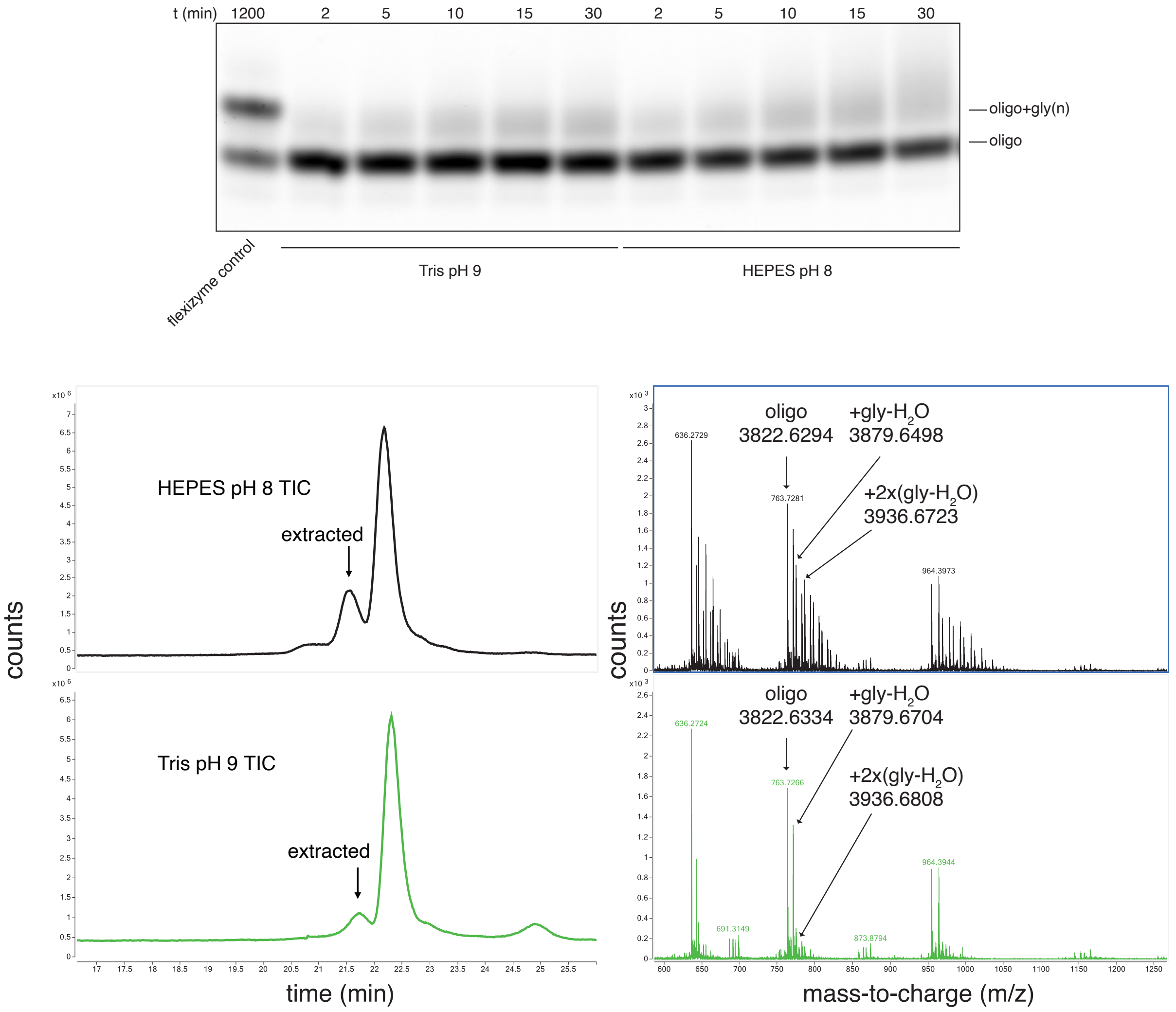

B

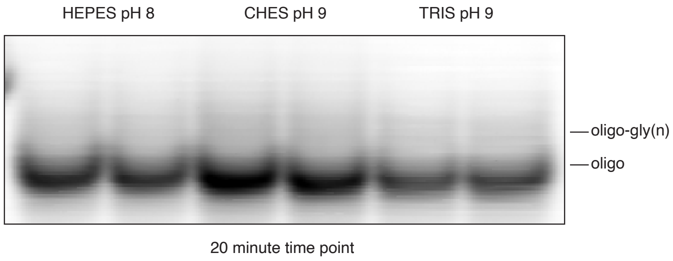

Figure S2. Aminoacylation of a model oligonucleotide. A (top): Acidic PAGE of a reaction timecourse between the model oligonucleotide (FX3 in Table S3) and CDI + glycine added at time point 0. The left-most lane contains a 1200-minute time point of the Flexizyme aminoacylation, which adds a single glycine on the 3′-terminus. **A** (bottom): Analysis of the same reaction by LC-TOF. The newly appearing signal in the total ion chromatogram (TIC) was extracted and exact masses were calculated for the labeled ions in the labeled charge envelope. Top left panel is the TIC of the aminoacylation reaction performed in HEPES pH 8. The top right panel is the extracted ion chromatogram (EIC) of the signal that appeared after the aminoacylation reaction. The exact masses were calculated for the labeled ions, and they corresponded to

the non-aminoacylated model oligonucleotide FX3 and the FX3 containing one or more glycyl esters. For clarity, only signals of the ions with the same charge are labeled. The bottom two panels are the same as the top ones, except the aminoacylation was performed in Tris pH 9. Under these conditions, only a singly glycylated FX3 can be observed. **B**: Acidic PAGE of the 20-minute time point of the same reaction as above, except the model oligonucleotide contained all internal deoxyribonucleotides, with only the 3′-terminal ribonucleotide (dFX3 in Table S3). The two lanes for each buffer condition represent two independent replicates. The two lanes for each buffer condition represent two independent replicates.

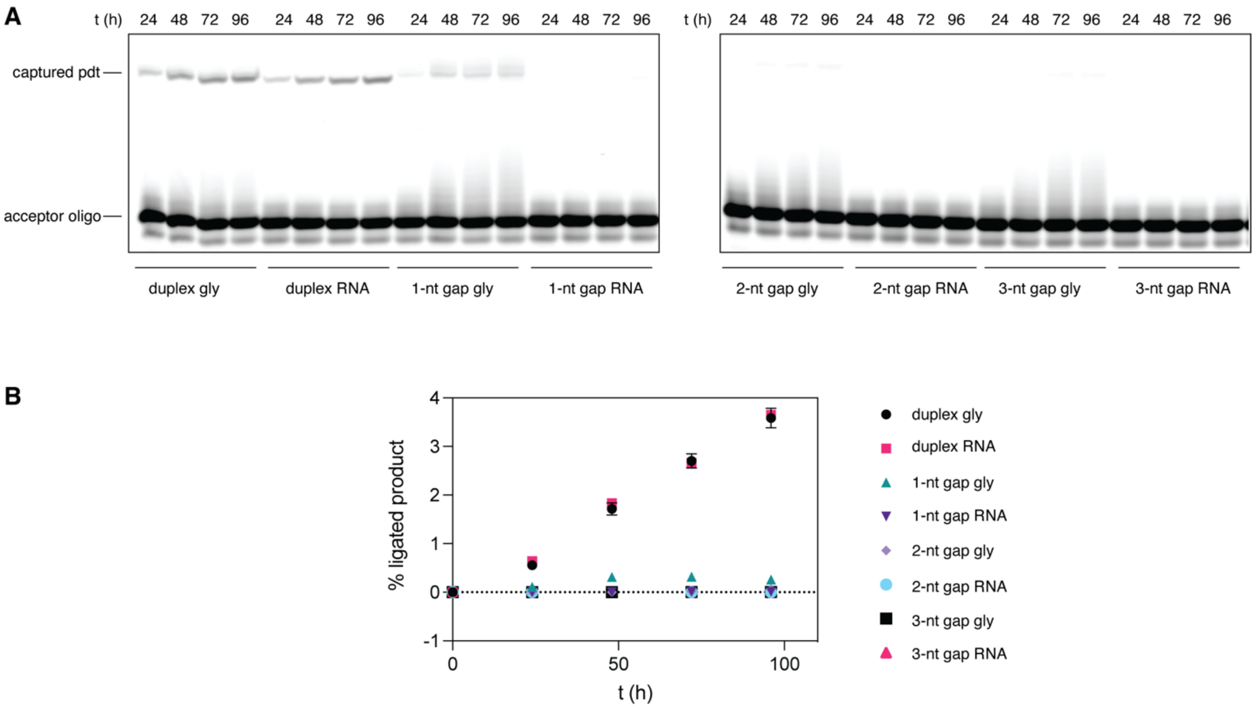

Figure S3. Activated amino acid capture by duplexed RNA. A: PAGE analysis of the time-course of the capture reaction with a nicked duplex and 1–3-nt gap-containing duplex. **B**: Quantification of the percent ligated or captured product from the gels in **A**. For each lane, the ratio of the ligated product band (top) to the sum of the ligated product and the starting material acceptor band (bottom) was determined and converted to percent product by multiplying it with 100 %.

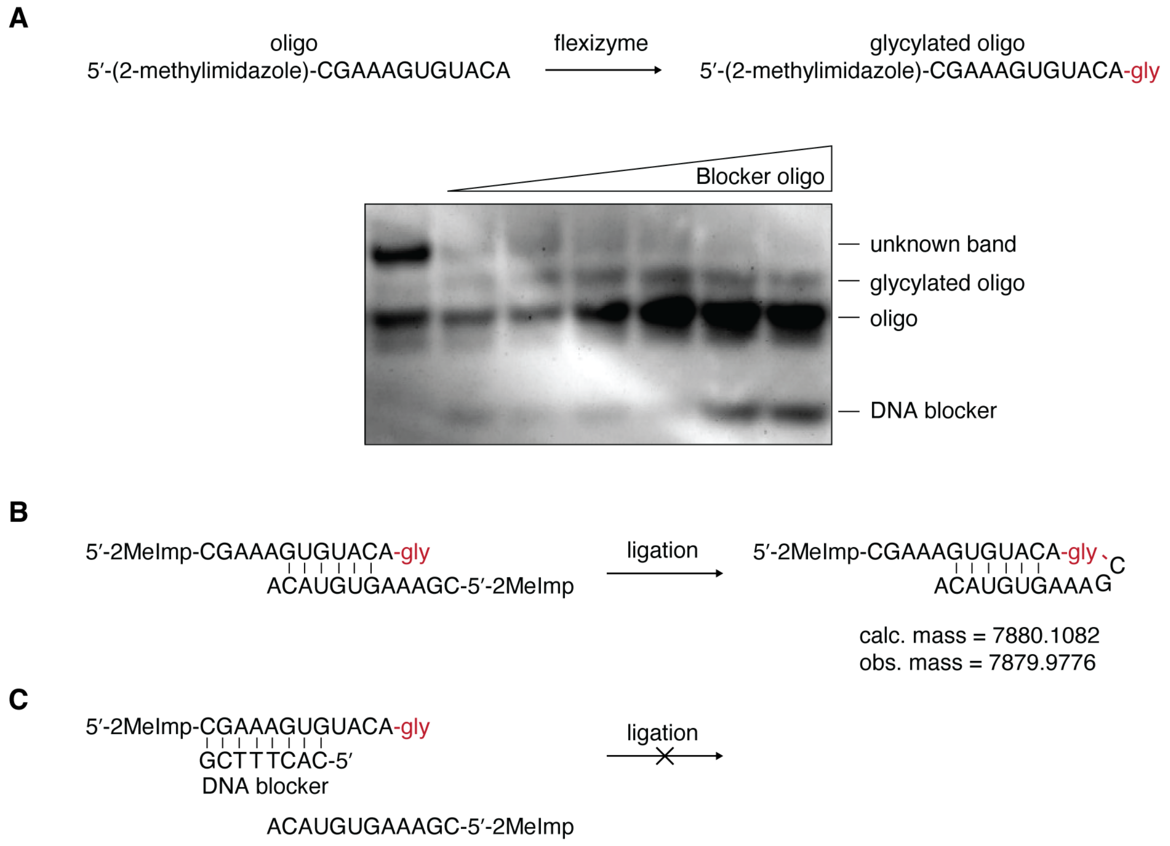

Figure S4. Flexizyme-catalyzed aminoacylation of a partially self-complementary oligonucleotide. **A**: The general reaction scheme and expected product. Acidic PAGE analysis of the reaction with and without an increasing concentration of a blocker oligonucleotide. Lane 1: aminoacylation reaction. Lanes 2–7: aminoacylation reaction in the presence of a DNA blocker at 10, 15, 20, 30, 40, and 50 μM, respectively. The gel was stained with SYBR Gold per manufacturer instructions. **B**: The hypothesized loop amino acid capture due to partial self-complementarity of the activated and aminoacylated oligonucleotide. LC-TOF analysis of the reaction confirming the appearance of the hypothesized product. **C**: Scheme of the blocker oligonucleotide preventing the amino acid-bridged loop formation.

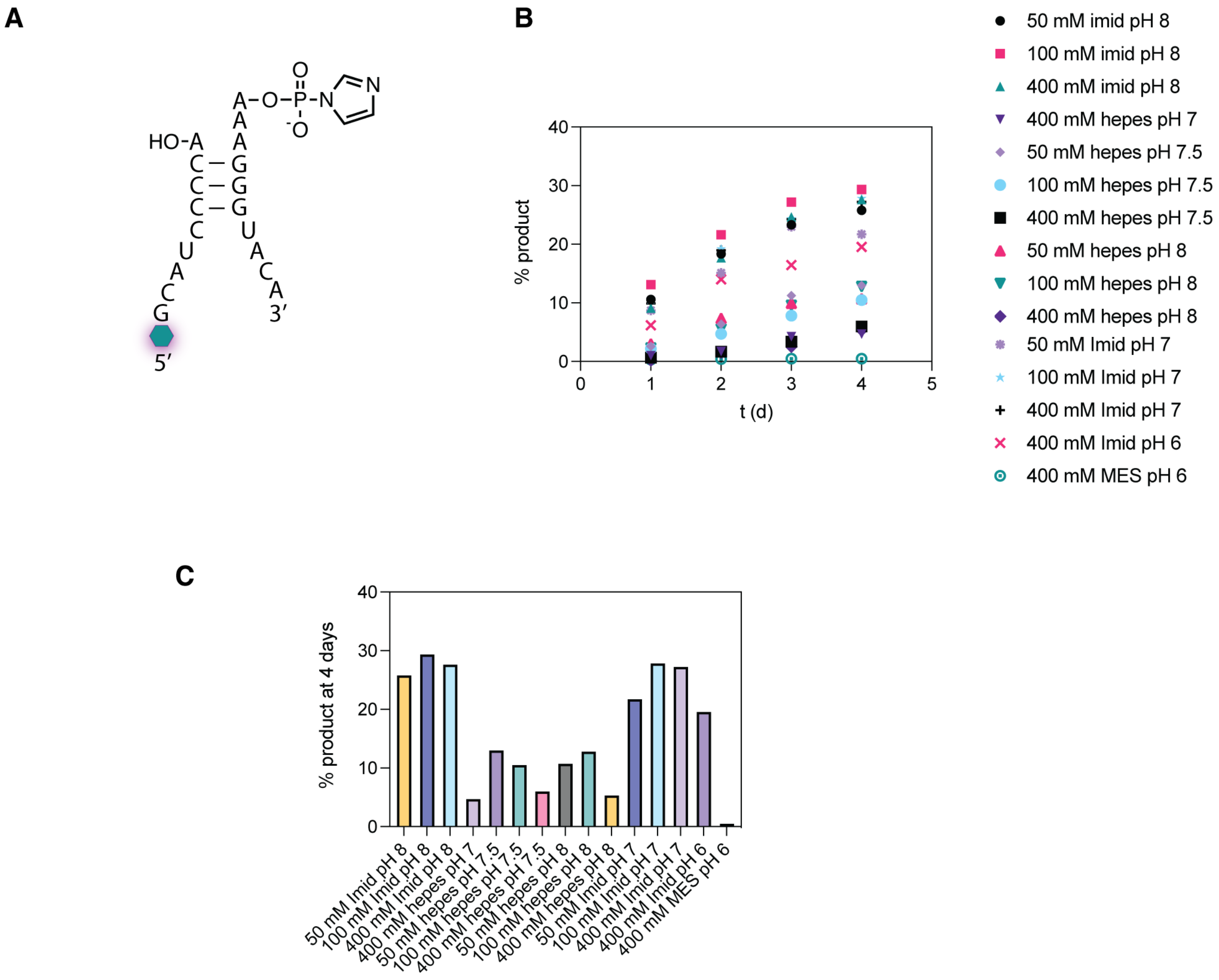

Figure S5. Optimization of the pH of the capture reaction using the modified P2 stem-loop of the Flexizyme. A: Diagram of the nicked stem-loop based on the P2 stem-loop found in the Flexizyme. **B**: Timecourse of the capture reaction at different concentrations of buffer and at different pHs. % product is quantified based on the per-lane normalized band intensity. **C**: Bar graph of the 4-day timepoint from **B** showing the maximum yield at each concentration and pH.

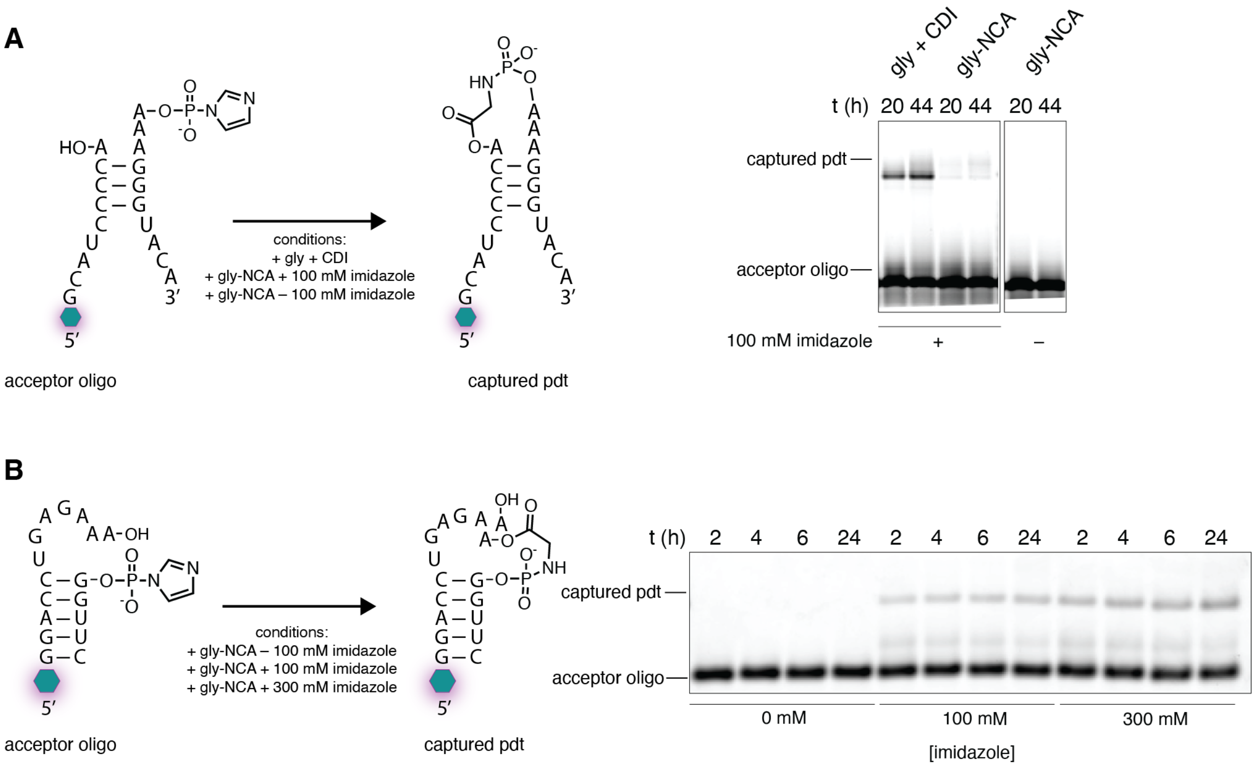

Figure S6. Imidazole-catalyzed gly-NCA capture using the modified P2 stem-loop of the Flexizyme and the T-loop containing RNA. A: On the left is the diagram of the capture reaction. On the right is PAGE analysis of the capture reaction. The conditions for the reactions were: 10 μM each oligonucleotide, 5 mM $MgCl_2$, 100 mM HEPES pH 8, and activated glycine as follows. Gly + CDI = 50 mM premixed gly and CDI; gly-NCA = presynthesized gly-NCA added instead of gly + CDI at 50 mM with or without 100 mM imidazole pH 8. **B**: On the left is the diagram of the capture reaction using the highly efficient T-loop containing RNA that we identified in our sequencing screen. Glycine is bridging the 2′-OH of the penultimate A of the 5′-UGAGAAA-3′ motif and the 5′-phosphate of the downstream G, as shown in Figure 3. On the right is PAGE analysis of the capture reaction. The conditions for the reactions were: 1 μM each oligonucleotide, 5 mM $MgCl_2$, 100 mM HEPES pH 8, and activated glycine as follows. 50 mM of the presynthesized gly-NCA was added to the reaction with the increasing concentration of imidazole pH 8.

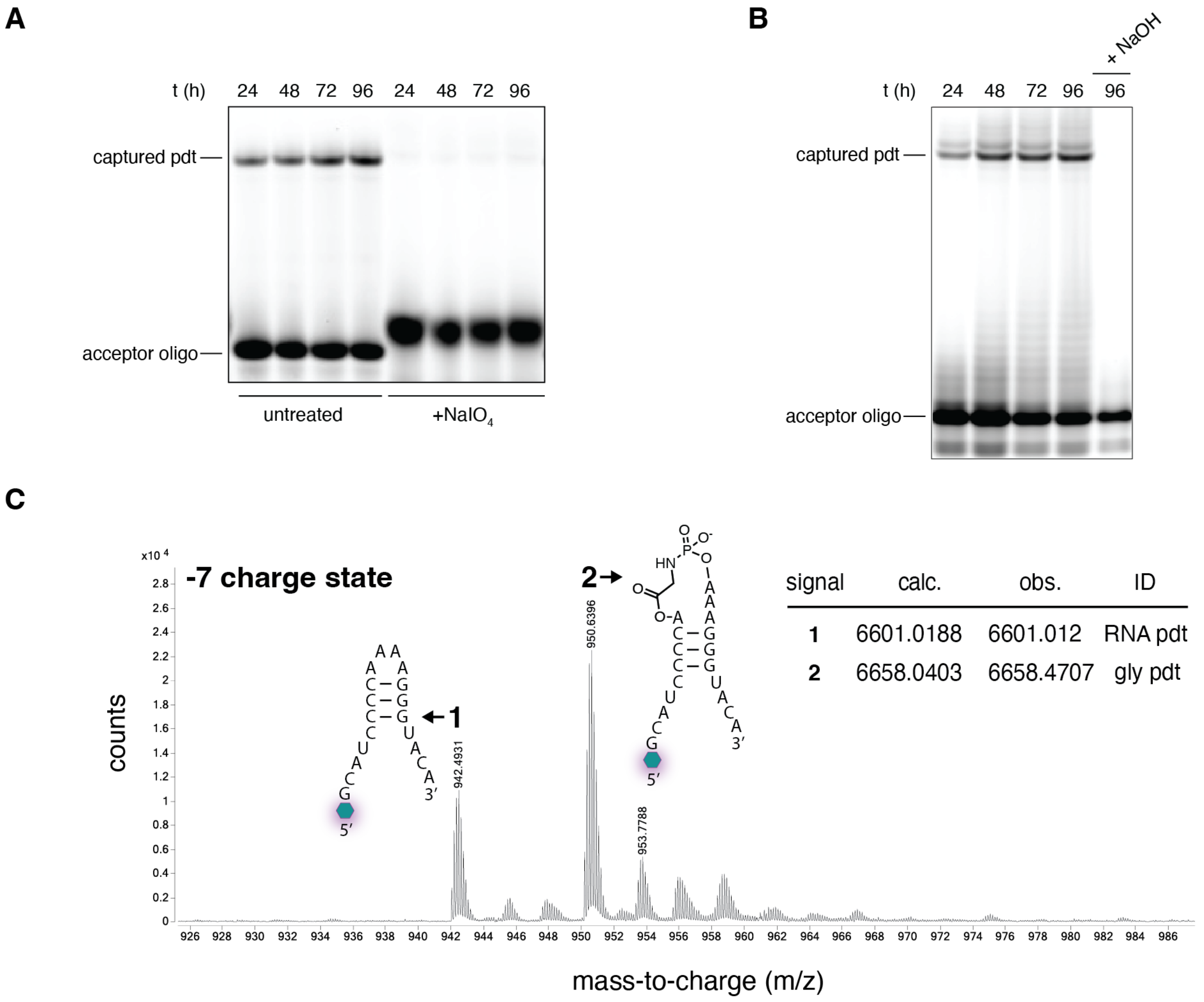

Figure S7. Loop-closing amino acid capture reaction characterization using the modified P2 stem-loop of the Flexizyme. A: PAGE analysis of the capture reaction with and without treatment of the acceptor strand with 10 mM $NaIO_4$. **B**: PAGE analysis of the capture reaction with the 96 h time point untreated and treated with 100 mM NaOH for 2 minutes. **C**: LC-TOF analysis of the crude 96 h reaction. Shown is the extracted ion chromatogram of –7 charge state of the reaction product. Signal 1, “RNA pdt”, is consistent with the product of the background RNA only reaction, which is beyond the detection limit of PAGE. Signal 2, “gly pdt”, is consistent with the glycine-bridged loop product.

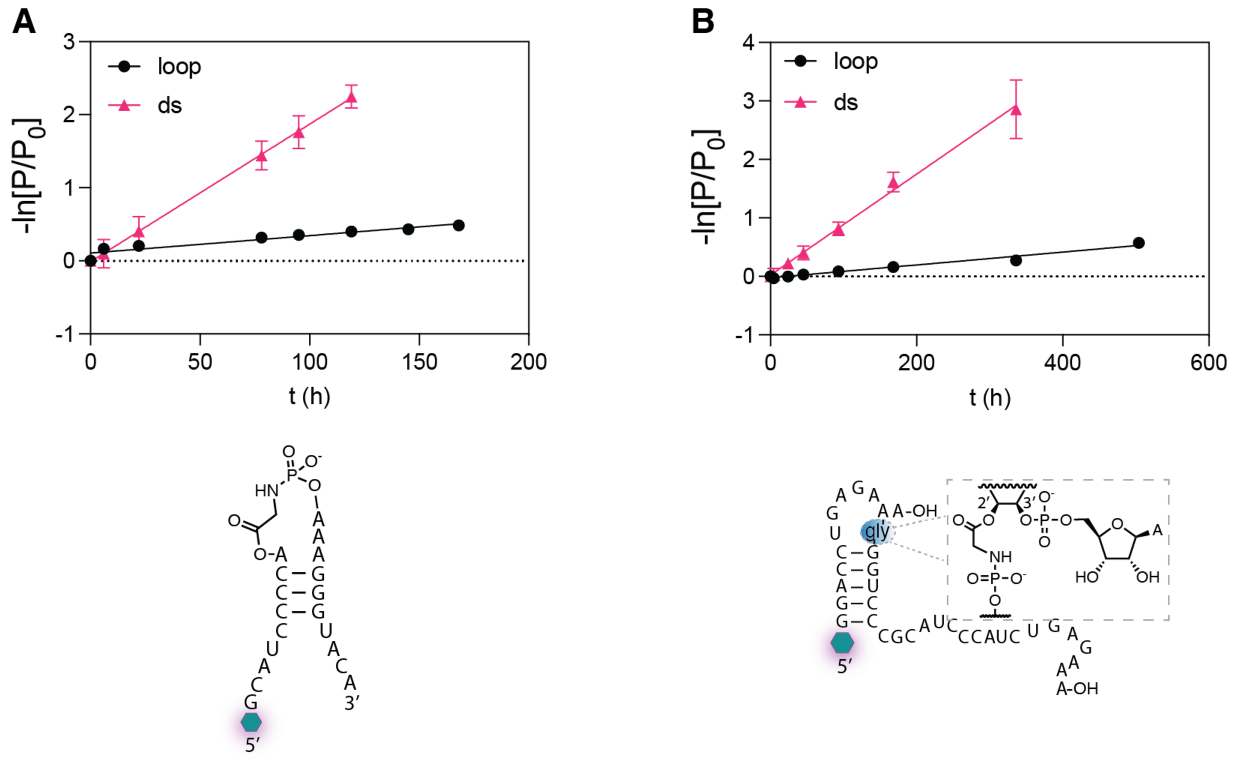

Figure S8. Kinetic plots of the glycine-bridged RNA degradation reactions. Negative natural logarithm of the ratio of the remaining product (P) to the initial product (P_0; assumed to be 1) at each time point. **A**: Hydrolysis of the glycine-bridged modified P2 stem-loop of the Flexizyme. Shown below the plot is the diagram of the loop structure. The structure was forced into the double stranded (ds) form by adding the complementary strand in 20-fold excess and performing an annealing cycle. **B**: Hydrolysis of the glycine-bridged modified P1 stem-loop of the Flexizyme containing the 5′-UGAGAAA-3′ T-loop motif. Shown below the plot is the diagram of the loop structure with glycine bridging the 2′-OH of the penultimate A of the 5′-UGAGAAA-3′ motif and the 5′-phosphate of the downstream G, as shown in Figure 3. The double stranded form was formed as above. Conditions: 200 mM HEPES pH 8, 2.5 mM $MgCl_2$, 0.75 μM annealed construct.

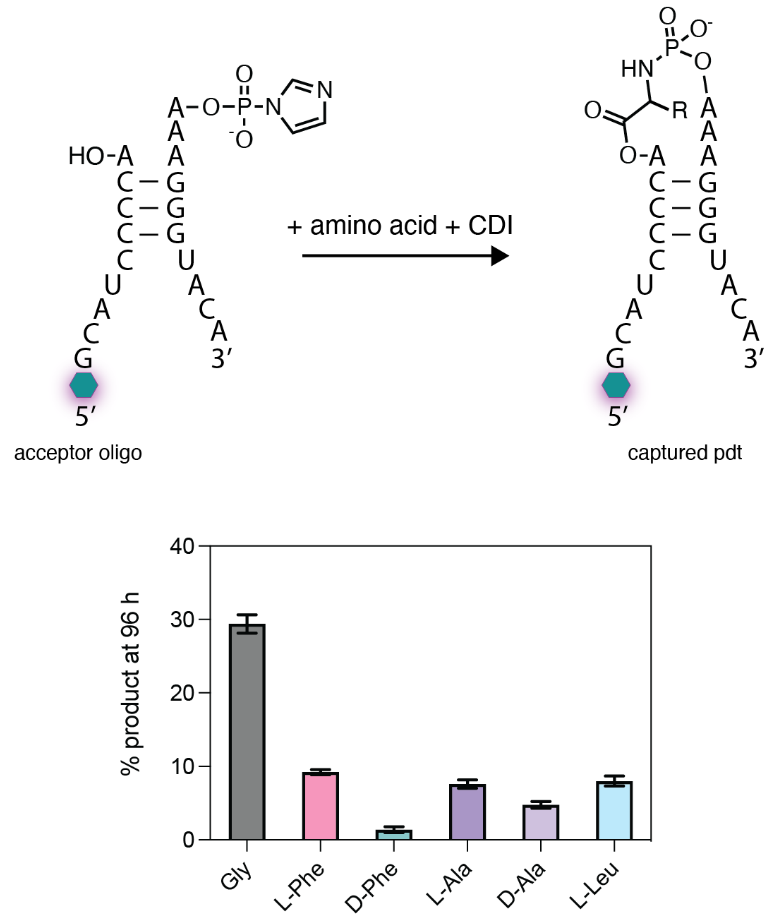

Figure S9. The capture reaction across amino acids for the modified P2 stem-loop of the Flexizyme. Shown is the final yield (at 96 hours) of the capture reaction, quantified by PAGE analysis based on the per-lane normalized band intensity. Standard conditions of 100 mM imidazole pH 8, 5 mM $MgCl_2$, 10 μM each oligonucleotide, 50 mM final concentration of premixed amino acid + CDI at 0 ºC were employed. CDI-activated glycine was added in four aliquots containing 0.25 μmol of the CDI-activated amino acid every 24 hours beginning at 0 hours. CDI activation was performed by adding 1.1 eq of CDI to 1 eq glycine in water every 24 hours.

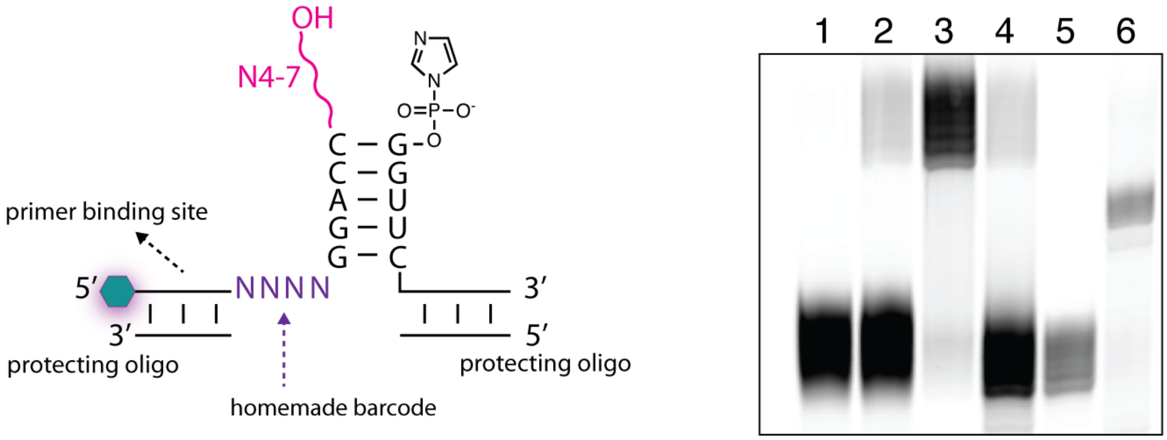

Figure S10. Schematic of the sequencing construct and quality controls. Nicked loop construct, containing a short 5-bp stem and a variable length (4-7 nt) randomized 3′-overhang (see Table S3 for the exact oligonucleotides). The glowing hexagon represents 5′-fluorescein. The encoded primer binding site allowed us to skip the ligation of the 5′-adapter. The protecting oligo was used to prevent any unwanted base-pairing interference. The acceptor oligonucleotide contained a homemade barcode to allow for facile filtering of sequences containing different overhang lengths during data analysis. On the right is a PAGE quality control analysis: lane 1 = reaction without gly showing no RNA-only product; lane 2 = reaction with gly + CDI; lane 3 = the product of the reaction with gly + CDI purified by PAGE; lane 4 = the purified product hydrolyzed with 100 mM NaOH for 3 minutes; lane 5 = PAGE-purified hydrolyzed product; lane 6 = RT primer binding site ligated to the PAGE-purified hydrolyzed product.

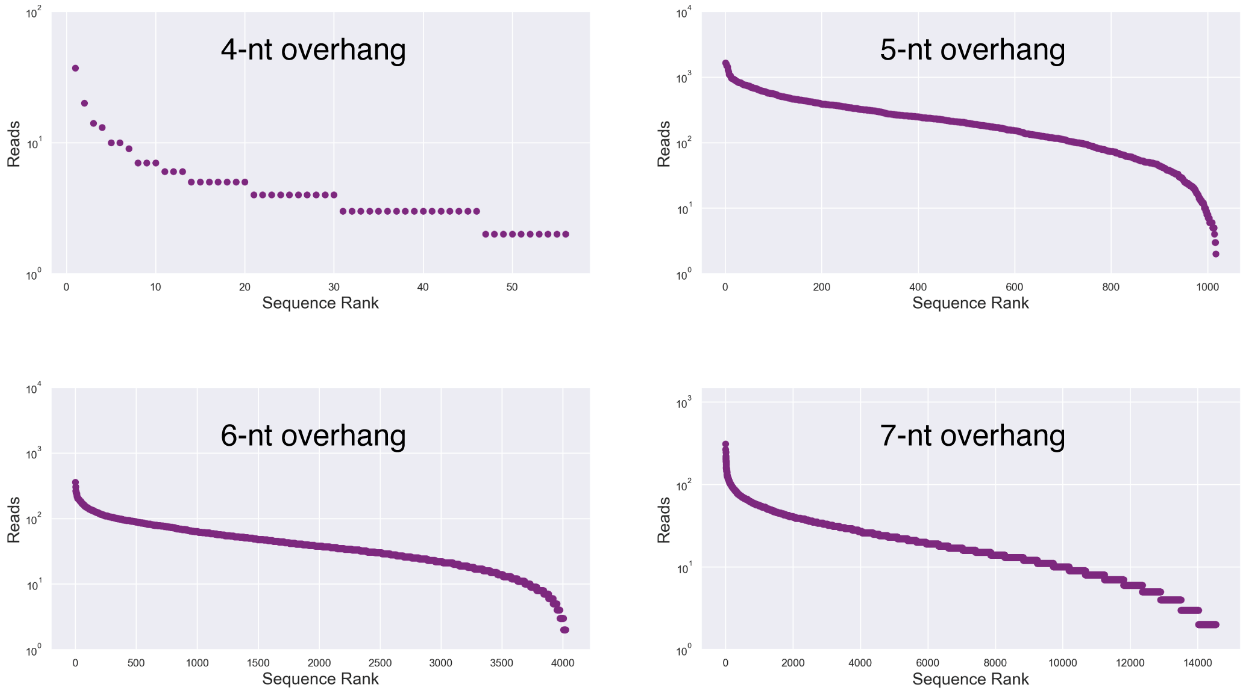

Figure S11. The distribution of sequencing reads across sequence space for the nicked stem-loop constructs. Filtered and sorted sequences plotted as Reads (log-scale) normalized to the distribution of reads in the starting library versus Sequence Rank. Each purple dot represents a unique sequence.

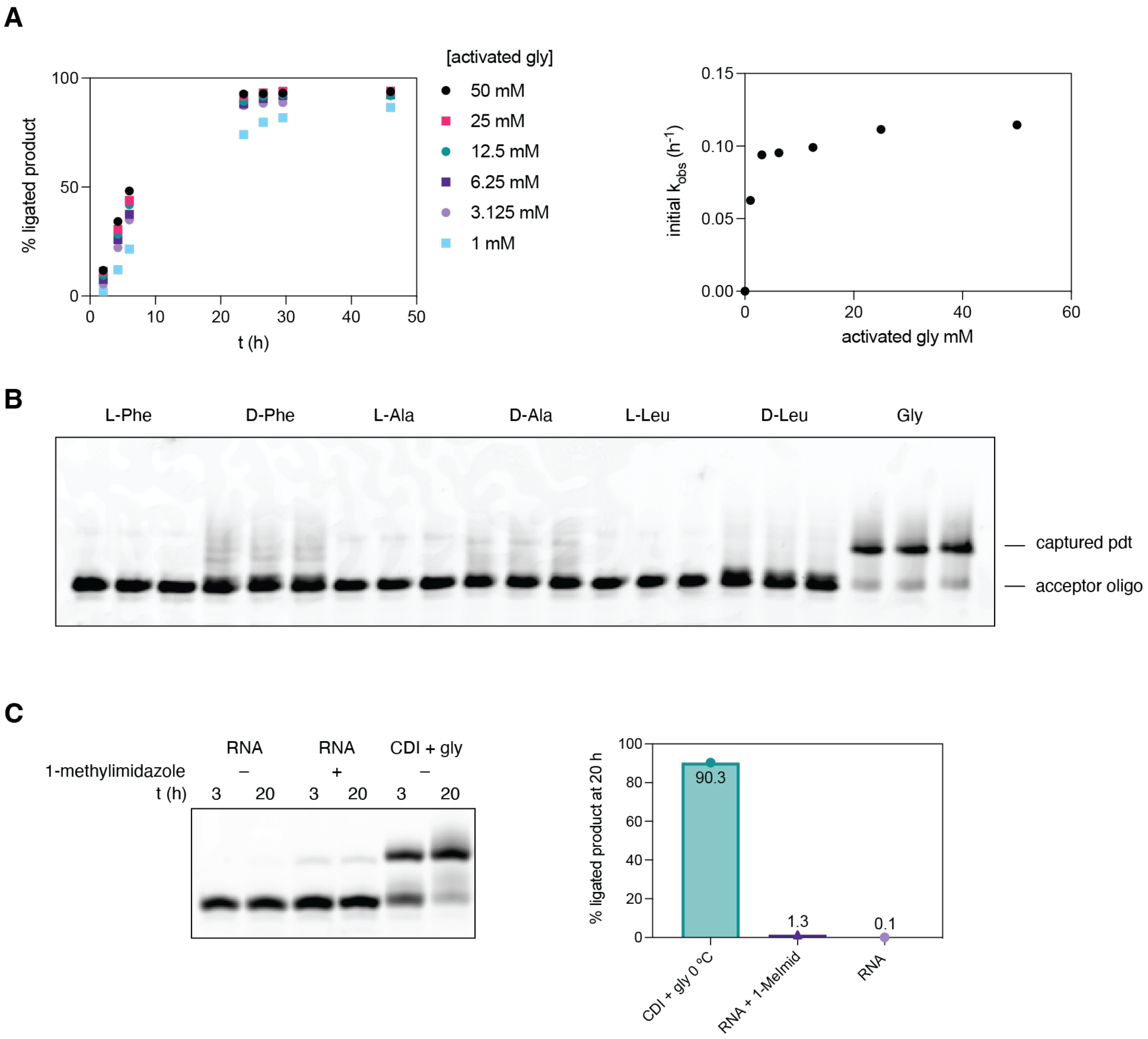

Figure S12. Capture of a variety of amino acids and ligation of RNA with the 5′-UGAGAAA-3′ nicked loop. A: On the left is the capture reaction yield over time with a serial dilution of the activated glycine under standard conditions. On the right is the plot of the initial observed rate constant for the capture reaction at the indicated activated glycine concentrations. The initial rates were obtained by plotting the negative natural logarithm of the percent remaining acceptor oligo for the first four time points of the reaction. **B**: PAGE analysis of the 96-hour time point of the capture reaction with a variety of activated amino acids. Each amino acid reaction was loaded in triplicates. **C**: RNA-only ligation using the UGAGAAA overhang and optimized RNA-only ligation conditions with and without the addition of the 1-methylimidazole catalyst. RNA-only conditions were 100 mM Na-HEPES pH 8, 50 mM $MgCl_2$, 1 µM each oligonucleotide, and 100 mM optional 1-methylimidazole at 22 ºC. The conditions for amino acid capture were 100 mM imidazole pH 8, 5 mM $MgCl_2$, 1 µM each oligonucleotide, and 50 mM amino acid + CDI at 0 ºC.

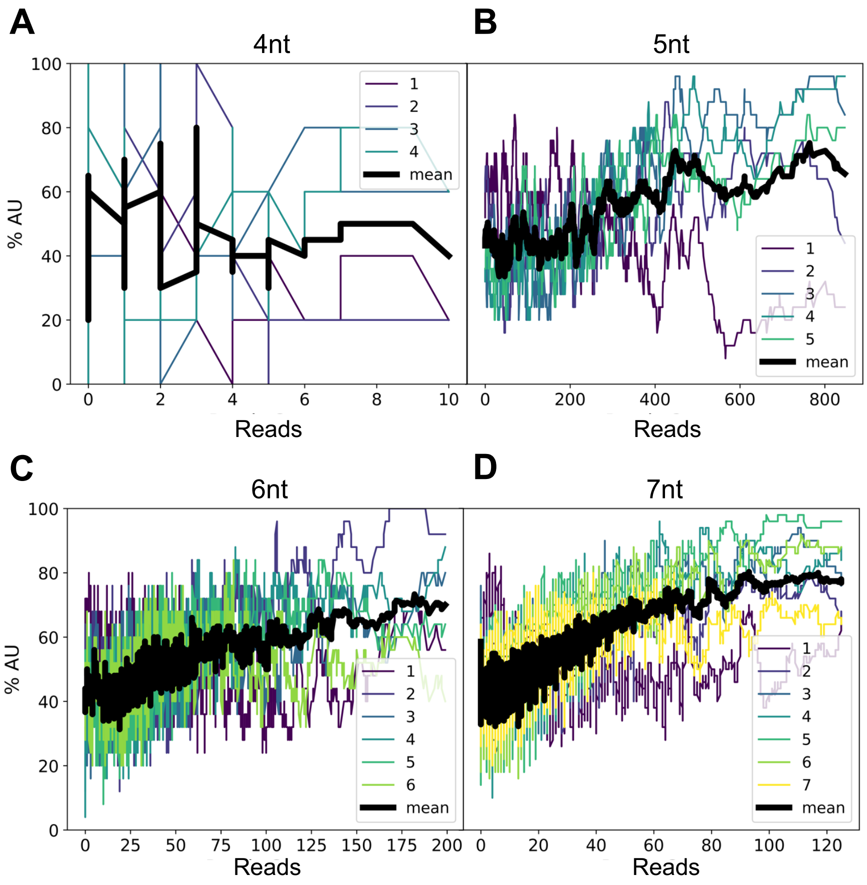

Figure S13. **AU-content as a function of reads for (A) 4-nt long loops, (B) 5-nt long loops, (C) 6-nt long loops, and (D) 7-nt long loops.** Colored traces show values calculated on a per-nucleotide basis, while the black trace shows their average. Every value is calculated by averaging the sorted sequences' read counts with the next 24 sequences (5-nt, 6-nt, and 7-nt long loops) or 4 sequences (4-nt long loops).

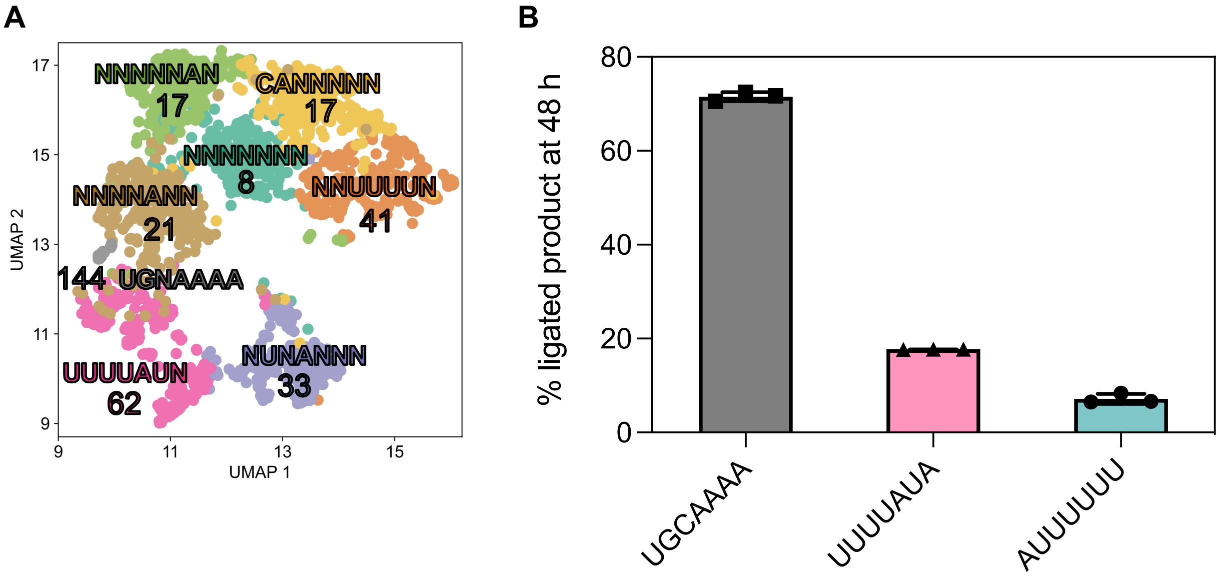

Figure S14. **Cluster analysis of the sequencing data using a K-means algorithm. A:** UMAP projection shows Shapley value space colored according to K-means clusters performed on high-dimensional data. A representative sequence for each cluster is shown here, with an arbitrary threshold of 0.35 for the frequency of a given nucleotide to be considered representative for a given position. Median reads for the cluster are also reported below the representative sequence. **B**: The capture reaction using a representative sequence from each of the top three clusters.

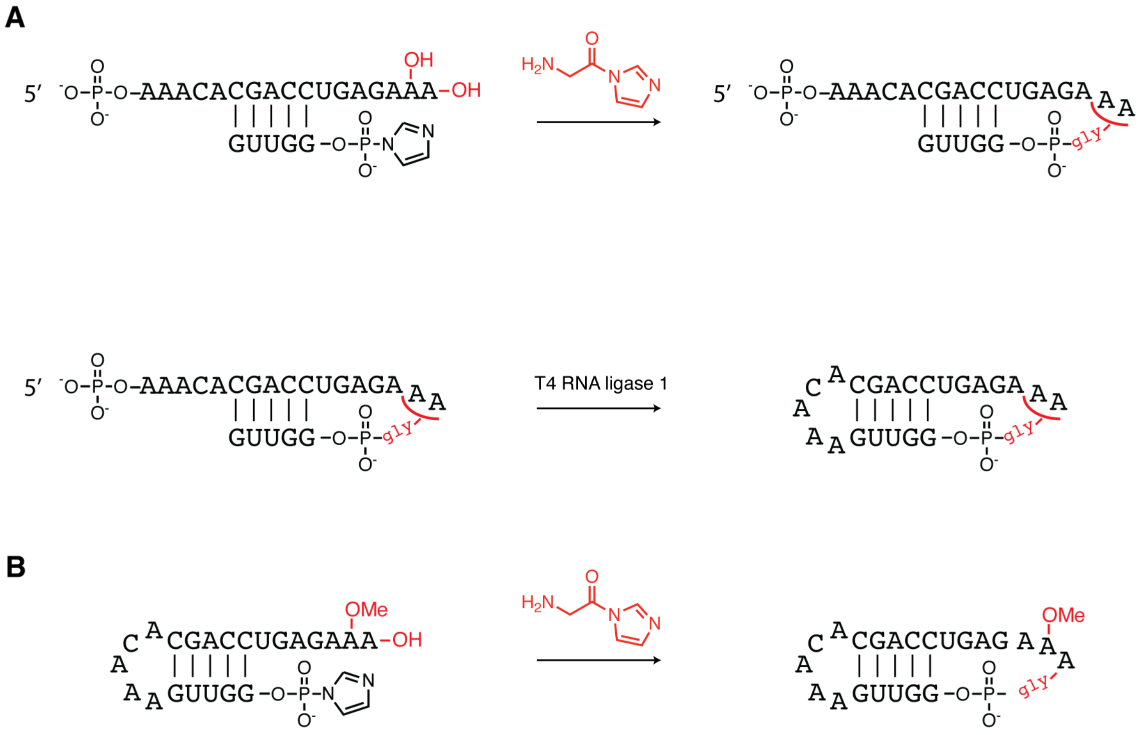

Figure S15. Synthesis of the dumbbell shaped RNAs for crystallography. A: Oligonucleotide with the UGAGAAA-3′ overhang is aminoacylated and ligated to the partially complementary oligonucleotide by incubation with CDI-activated gly. The 5′-AAACA overhang is then closed into a loop with T4 RNA ligase 1. **B**: Oligonucleotide that already has the 5′-AAACA preinstalled during synthesis is closed on the UGAGAmAA-3′ end by incubation with CDI-activated gly.

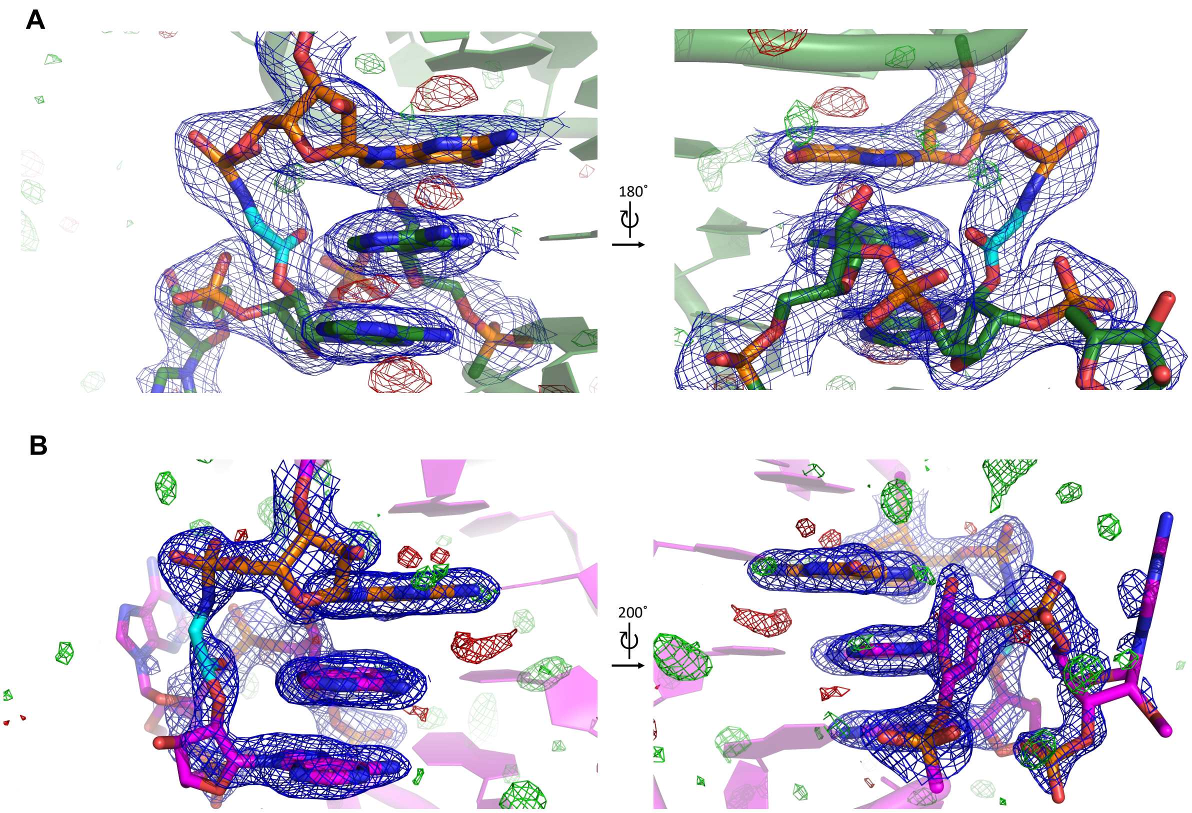

Figure S16. Electron density maps corresponding to the glycine linker and residues G1, A20, A21 and A22 in both crystallized constructs. Blue meshes represent the 2Fo − Fc electron density map at 1.5σ contour level and carve radius 2 Å. Fo − Fc map represented as green mesh at + 3.0 σ and red at − 3.0 σ). **A**: Loop-closed dumbbell RNA bridged by glycine. **B**: Loop-closed A21 2′-OMe dumbbell RNA bridged by glycine.

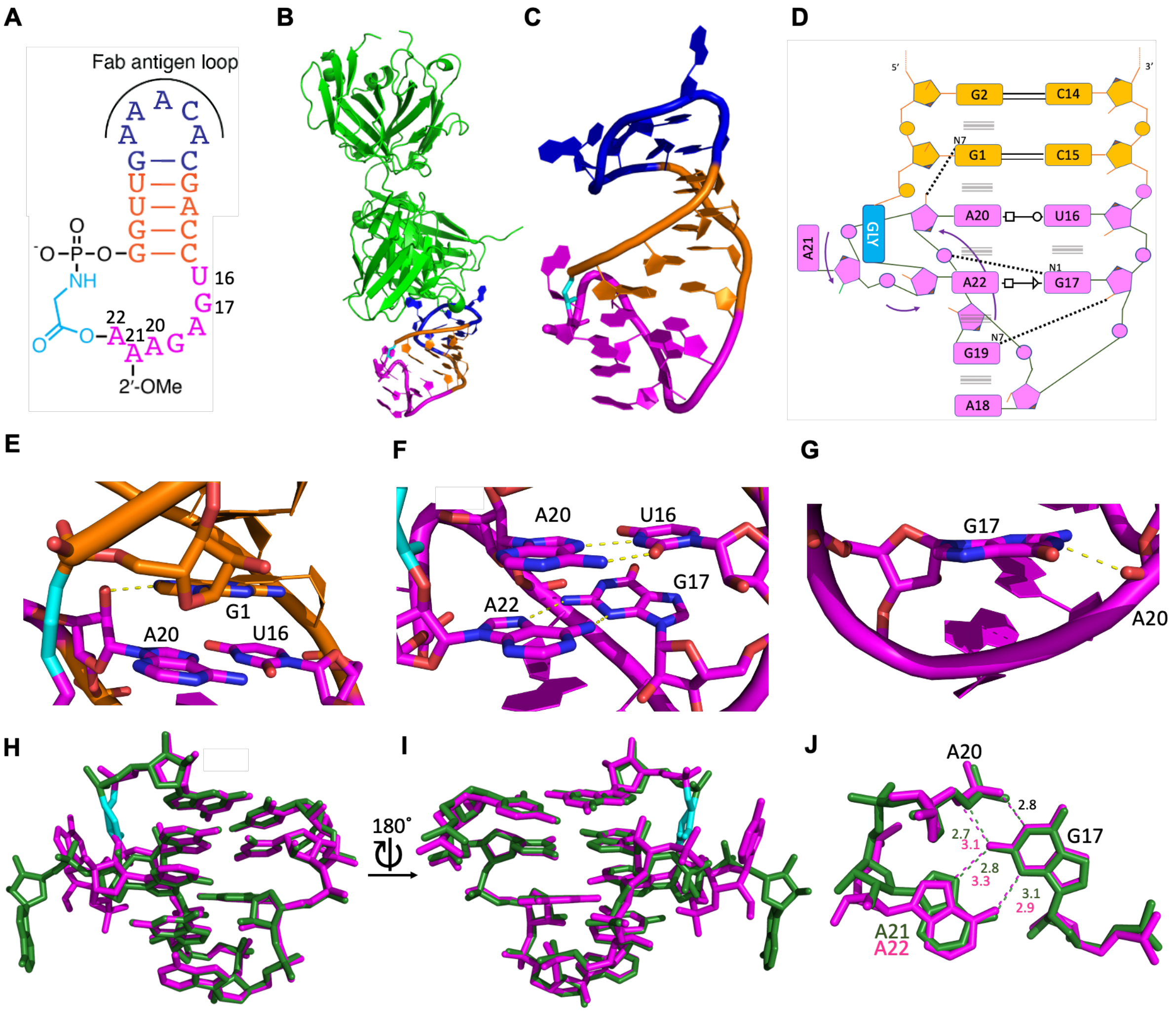

Figure S17. Secondary and tertiary structures of the loop-closed A21 2′-OMe dumbbell RNA bridged by glycine determined by crystal structures. A: Schematic secondary structure of the 2′-OMe modified chimeric dumbbell RNA crystallization construct. **B:** Overall arrangement of loop-closed A21 2′-OMe dumbbell RNA bridged by glycine and Fab BL3-6 in the crystal structure, the closed loop in magenta, glycine linker in cyan. **C:** Tertiary structure of the loop-closed construct. **D:** Secondary structure of the closed loop, interactions are denoted using the Leontis-Westhof nomenclature. **E:** A20:2′OH interacts with G1:N7. **F:** Parallel base pairing interactions in the ligated loop. **G:** G17:N1 interacts with A20:OP2. **H:** Superposition of structures derived from the unmodified dumbbell closed loop (green) and the A21 2′-OMe dumbbell closed loop (magenta), glycine linker in cyan in both structures. **I:** Same as G but rotated 180 in y axis. **J:** Distances in Angstrom and possible interactions (dashed lines) between G17, A20 and intercalating A in the ligated loop crystal structures.

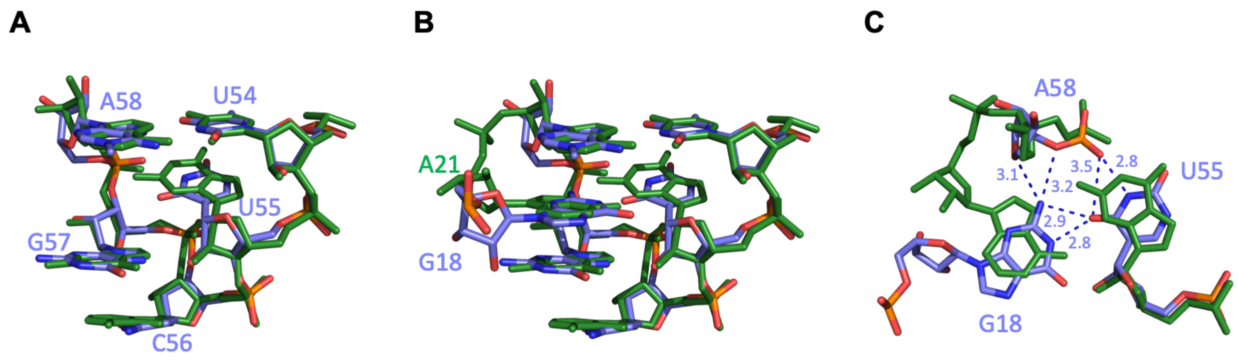

Figure S18. The overlay of the tertiary structure of the dumbbell RNA bridged by glycine and the tRNAphe T-loop. A: Superposition of the closed loop structural motif and the T-loop motif (light blue) from the tRNAphe (PDB 1EHZ) without the intercalating base. **B**: Superposition of the closed loop structural motif and the T-loop motif (light blue) from the tRNAphe (PDB 1EHZ), together with intercalating bases. **C**: Distances and interactions between G18 and U55 and A58 of tRNAphe (PDB 1EHZ) superimposed with the closed glycine-bridged loop (green).

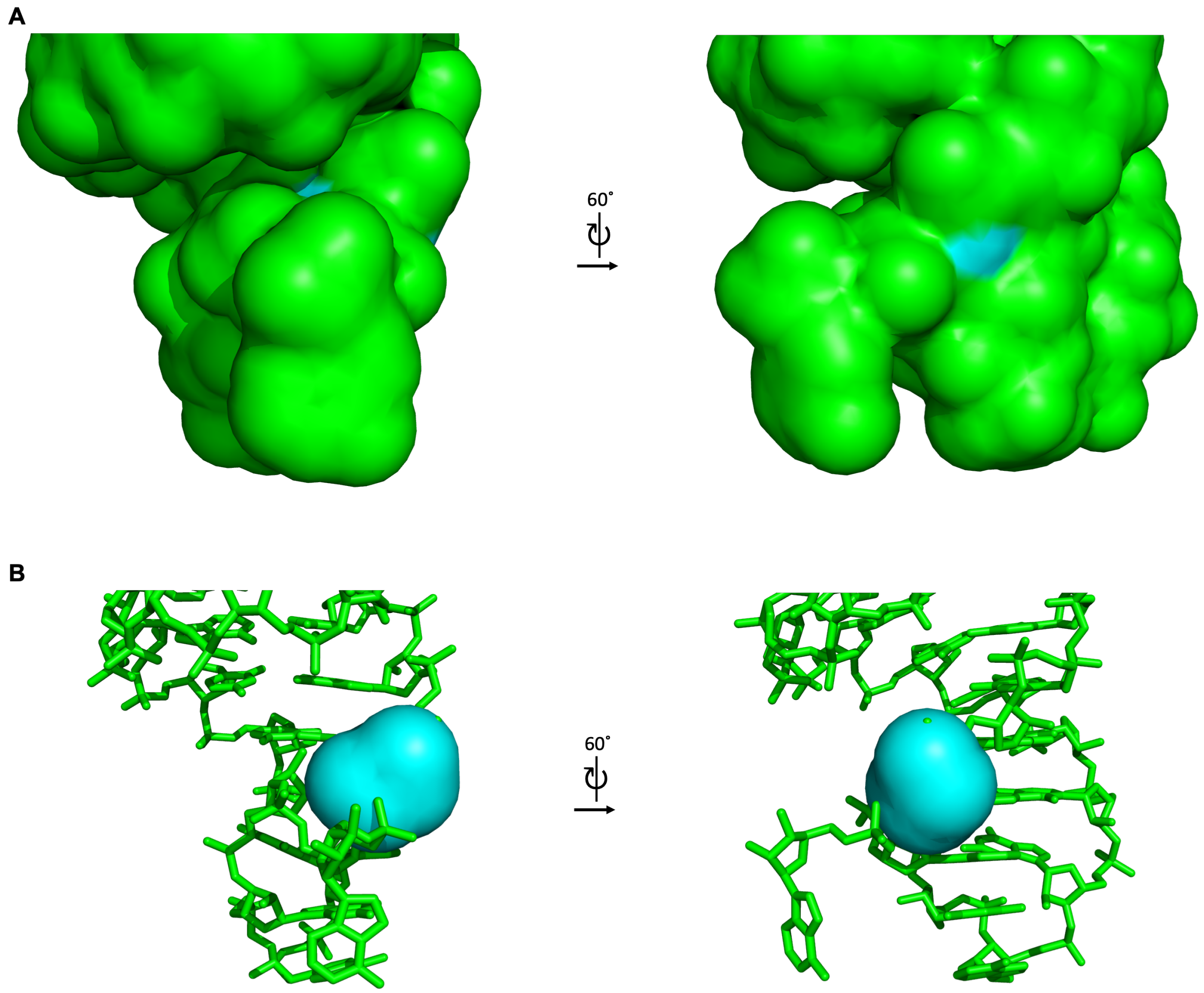

Figure S19. Solvent-accessible surface area (SASA) of the glycine-bridged dumbbell RNA. Figures were generated in PyMOL with the solvent radius probe of 1.4 Å. **A:** SASA presented as solid surface for RNA in green and the glycine bridge in cyan. **B:** SASA presented as solid cyan surface for the glycine bridge only.

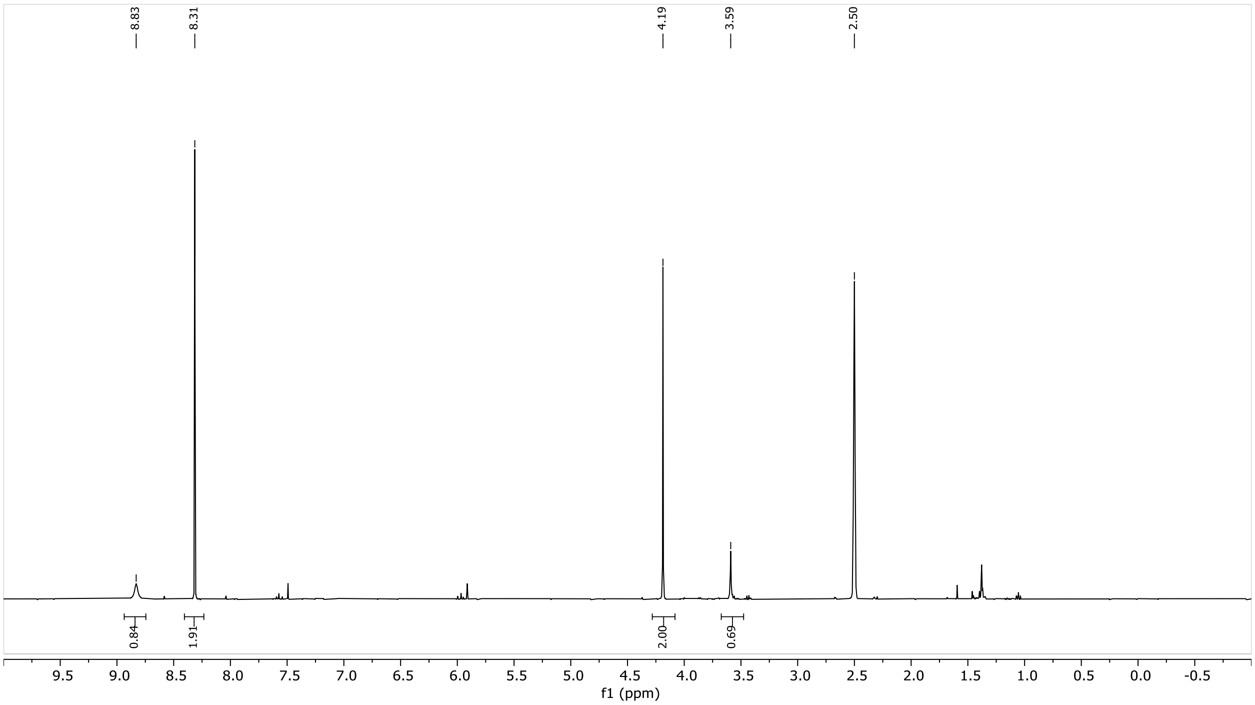

Figure S20. ^{1}H NMR spectrum of the crude synthesis reaction of Gly-NCA. Glycine-NCA: ^{1}H NMR (400 MHz, dmso) δ 8.83 (s, 1H), 4.19 (s, 2H). The ^{1}H NMR spectral data matched with previously reported values for Glycine-NCA(18, 19). Glycine.HCl and Glycine.PO(OH)$_3$: ^{1}H NMR (400 MHz, dmso) δ 8.31 (s, ~5H), 3.59 (s, 2H). The large number of protons in the signal at 8.31 is likely stemming from a mixture of the two glycine salts.

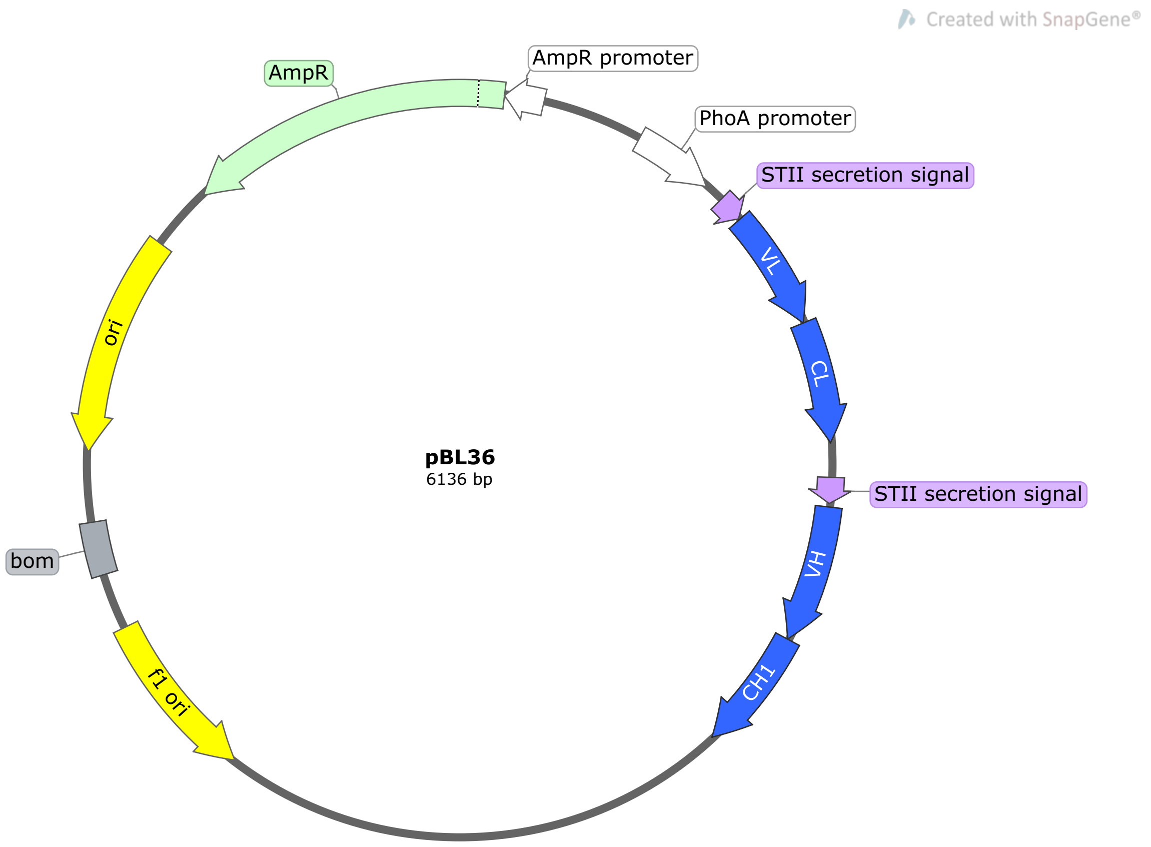

STII signal peptide and Fab Light chain

MKKNIAFLLASMFVFSIATNAYASDIQMTQSPSSLSASVGDRVTITCRASQSVSSAVAWYQQKPGKAPKLLIYSASSLYSG
VPSRFSGSRSGTDFTLTISSLQPEDFATYYCQQSYSFPSTFGQGTKVEIKRTVAAPSVFIFPPSDEQLKSGTASVVCLLNN
FYPREAKVQWKVDNALQSGNSQESVTEQDSKDSTYSLSSTLTLSKADYEKHKVYACEVTHQGLSSPVTKSFNRGEC*

STII signal peptide and Fab Heavy chain

MKKNIAFLLASMFVFSIATNAYAEISEVQLVESGGGLVQPGGSLRLSCAASGFYISYSSIHWVRQAPGKGLEWVASISPY
SGSTYYADSVKGRFTISADTSKNTAYLQMNSLRAEDTAVYYCARQGYRRRSGRGFDYWGQGTLVTVSSASTKGPSVFPL
APSSKSTSGGTAALGCLVKDYFPEPVTVSWNSGALTSGVHTFPAVLQSSGLYSLSSVVTVPSSSLGTQTYICNVNHKPS
NTKVDKKVEPKSCDKTHT*

Figure S21. BL3-6 Fab expression vector. Top: plasmid BL3-6 map generated with SnapGene. Bottom: Fab chains with their signal peptides underlined.

Table S1.

Stability of the aminoacyl ester linkage in different sequence and structure contexts. Conditions: 200 mM HEPES pH 8, 2.5 mM $MgCl_2$ at 22 °C. [a]value taken from ref. (8) for sequence A4C4 in Table S3. The uncertainty represents the 95 % confidence interval based on three replicates.

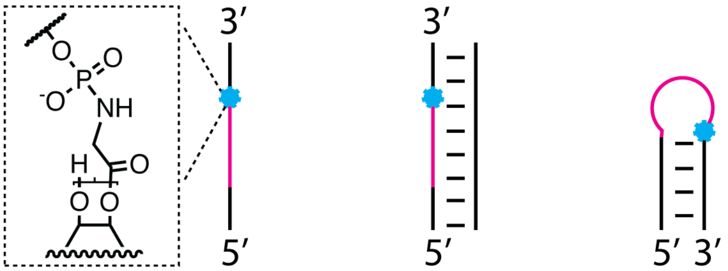

	Half life (h^{-1})		
Sequence	ss	ds	loop
AAAA	–	37 ± 3	290 ± 43
UGAGAAA	–	80 ± 6	631 ± 52
GACA[a]	20 ± 1[a]	72 ± 2[a]	–

Table S2.

Data collection and refinement statistics. Statistics for the highest-resolution shell are shown in parentheses.

	**Loop-closed dumbbell RNA bridged by glycine**	**Loop-closed A21 2′-OMe dumbbell RNA bridged by glycine**
**Wavelength**	0.979180	0.979180
**Resolution range**	46.07 - 2.07 (2.144 - 2.07)	41.56 - 1.57 (1.626 - 1.57)
**Space group**	P 1	P 1 21 1
**Unit cell**	38.514 82.085 92.668 63.953 88.687 88.401	39.466 83.126 73.339 90 90.255 90
**Total reflections**	222414 (22208)	221740 (20512)
**Unique reflections**	57272 (5776)	65261 (6414)
**Multiplicity**	3.9 (3.8)	3.4 (3.2)
**Completeness (%)**	91.14 (91.81)	96.43 (98.30)
**Mean I/sigma(I)**	6.55 (1.12)	9.31 (1.48)
**Wilson B-factor**	39.78	19.94
**R-merge**	0.136 (0.9437)	0.6197 (0.7562)
**R-meas**	0.1576 (1.096)	0.7276 (0.9121)
**R-pim**	0.07945 (0.5563)	0.378 (0.5034)
**CC1/2**	0.992 (0.591)	0.903 (0.488)
**CC***	0.998 (0.862)	0.974 (0.81)
**Reflections used in refinement**	56650 (5775)	63650 (6414)
**Reflections used for R-free**	2025 (195)	1993 (199)
**R-work**	0.2211 (0.3020)	0.1861 (0.2677)
**R-free**	0.2537 (0.3368)	0.2122 (0.2799)
**CC(work)**	0.953 (0.750)	0.959 (0.756)
**CC(free)**	0.964 (0.651)	0.946 (0.602)
**RMS(bonds)**	0.003	0.006
**RMS(angles)**	0.58	0.93

**Ramachandran favored (%)**	97.25	97.95
**Ramachandran allowed (%)**	2.75	2.05
**Ramachandran outliers (%)**	0.00	0.00
**Rotamer outliers (%)**	0.93	0.26
**Clashscore**	3.84	3.00
**Average B-factor**	51.11	27.23

Table S3.

Sequences used in this work. Nucleotides in purple are deoxyribonucleotides, while all others are ribonucleotides. 2-MeImp represents 2-methylimidazole activated phosphate. Imp represents imidazole activated phosphate. Prefix “m” represents 2′-O-methyl nucleotides. “(circ)” indicates the sites of circularization.

Name	Sequence	Use
A1	5′-fluorescein-AGAGAGCAGACA	Figures 1B, S3
C1	5′-Imp-CCCGGCAGCU	Figures 1B, S3
T1	5′-AGCUGCCGGGAUGUCUGCUCUCU	Figures 1B, S3
T2	5′-AGCUGCCGGGAAUGUCUGCUCUCU	Figure S3
T3	5′-AGCUGCCGGGAAAUGUCUGCUCUCU	Figure S3
A2	5′-fluorescein-GCAUCCCCA	Figures 1C, S5,6,7,9
C2	5′-Imp-AAAGGGUACA	Figures 1C, S5,6,7,9
FX3	5′-fluorescein-AGAGAAGCCA	Figure S2A
dFX3	5′-fluorescein-AGAGAAGCCA	Figure S2B
Pc2_m4	5′-2-MeImp-CGAAAGUGUACA	Figure S4
Block	5′-CACTTTCG	Figure S4C
A2C2	5′-fluorescein-GCAUCCCCA-gly-AAAGGGUACA	Table S1, Figure S8A
A2C2duplex	5′-UGUACCCUUUUGGGGAUGC	Table S1, Figure S8A
A2C2revcomp	5′-GCAUCCCCAAAAGGGUACA	Table S1, Figure S8A
A3C3	5′-fluorescein-GGACCUGAGAAA-gly-GGUCCCGCAUCCCAGUCUGAGAAA	Table S1, Figure S8B
A3C3duplex	5′-GAGAAUAACGCCAUGUACCAGUCUUUCUC GACUGGGAUGCGGGACCUUUCUCAGGUCC	Table S1, Figure S8B
A3C3revcomp	5′-GGACCUGAGAAAGGUCCCGCAUCCCAGUCUGAGAAAGACUGGUACAUGGCGUUAUUCUC	Table S1, Figure S8B
A4C4	5′-fluorescein-AGAGAAGAGAGCAGACA-gly-CCCGGCAGCU	Table S1, ref. 8
A5	5′-fluorescein-GUUCAGAGUUCUACAGUCCGACGAUCGCGAGGACCNNNN	Figures 2, S10, S11, S13

A6	5′-fluorescein-GUUCAGAGUUCUACAGUCCGACGAUCUUCAGGACCNNNNN	Figures 2, S10, S11, S13
A7	5′-fluorescein-GUUCAGAGUUCUACAGUCCGACGAUCCAUCGGACCNNNNNN	Figures 2, S10, S11, S13
A8	5′-fluorescein-GUUCAGAGUUCUACAGUCCGACGAUCACAAGGACCNNNNNNN	Figures 2, S10, S11, S13-14
C5	5′-Imp-GGUUCAAUAAGAUCGGAAGAGCACACGUCU	Figures 2, S10
RT_3′protector	5′-AGACGTGTGCTCTTCCGATCT	Figures 2, S10
5′protector	5′-GATCGTCGGACTGTAGAACTCTGAAC	Figures 2, S10
3′-RT_binding	5′-AppAGATCGGAAGAGCACACGTCT/3ddC	Figures 2, S10
A9	5′-fluorescein-GGACCGCGC	Figure 2B
A10	5′-fluorescein-GGACCGCAA	Figure 2B
A11	5′-fluorescein-GGACCCGGAU	Figure 2B
A12	5′-fluorescein-GGACCCGGAA	Figure 2B
A13	5′-fluorescein-GGACCAUUUUG	Figure 2B
A14	5′-fluorescein-GGACCAUUUCG	Figure 2B
A15	5′-fluorescein-GGACCUGUGAAA	Figure 2B
A16	5′-fluorescein-GGACCUGCGAAA	Figure 2B
A17	5′-fluorescein-GGACCUGAGAAA	Figures 2, 4, 5, S12
C6	5′-Imp-GGUUC	Figures 2B, 4, S12, S14B
A56	5′-phosphate-AAACACGACCUGAGAAA	Figure S15A
C9	5′-Imp-GGUUG	Figure S15A
chimeric dumbbell RNA	(circ)AAACACGACCUGAGAAA-gly-GGUUG(circ)	Figures 3, S15A, S16A, S17H-J, S18, S19
A57	5′-Imp-GGUUGAAACACGACCUGAGAmAA	Figures S15B
A21 2′-OMe chimeric dumbbell RNA	(circ)AAACACGACCUGAGAmAA-gly-GGUUG(circ)	Figures S16B, S17
A17–	5′-fluorescein-GGACCUGAGAA	Figure 4

A18	5′-fluorescein-GGACCUGAGAmAA	Figure 4
A19	5′-fluorescein-GGACCUIAGAAA	Figure 4
A20	5′-fluorescein-GGACCUGAGA2ApA	Figure 4
A21	5′-fluorescein-GGACCUUUUAUA	Figure S14B
A22	5′-fluorescein-GGACCAUUUUUU	Figure S14B
Piece 1 (A17)	5′-fluorescein-GGACCUGAGAAA	Figure 5
Piece 2	5′-Imp-GGUCCCGCAUCCCAUCUGAGAAA	Figure 5
Piece 3	5′-Imp-GAUGGUACAUGGCGUUAUUCUC	Figure 5
RNA dFx_S7	5′-fluorescein-GGACCUGAGAAAGGUCCCGCAUCCCAUCUGAGAAAGAUGGUACAUGGCGUUAUUCUC	Figure 5C
Chimeric dFx_S7	5′-fluorescein-GGACCUGAGAAA-gly-GGUCCCGCAUCCCAUCUGAGAAA-gly-GAUGGUACAUGGCGUUAUUCUC	Figure 5C
Substrate oligo (A17)	5′-fluorescein-GGACCUGAGAAA	Figure 5C
